# Unraveling the Ultrafast Photochemical Dynamics of Nitrobenzene in Aqueous Solution

**DOI:** 10.1021/jacs.3c13826

**Published:** 2024-04-04

**Authors:** Nicholas A. Lau, Deborin Ghosh, Susannah Bourne-Worster, Rhea Kumar, William A. Whitaker, Jonas Heitland, Julia A. Davies, Gabriel Karras, Ian P. Clark, Gregory M. Greetham, Graham A. Worth, Andrew J. Orr-Ewing, Helen H. Fielding

**Affiliations:** † Department of Chemistry, University College London, 20 Gordon Street, London WC1H 0AJ, U.K.; ‡ School of Chemistry, University of Bristol, Cantock’s Close, Bristol BS8 1TS, U.K.; § Central Laser Facility, Research Complex at Harwell, STFC Rutherford Appleton Laboratory, Didcot, Oxfordshire OX11 0QX, U.K.

## Abstract

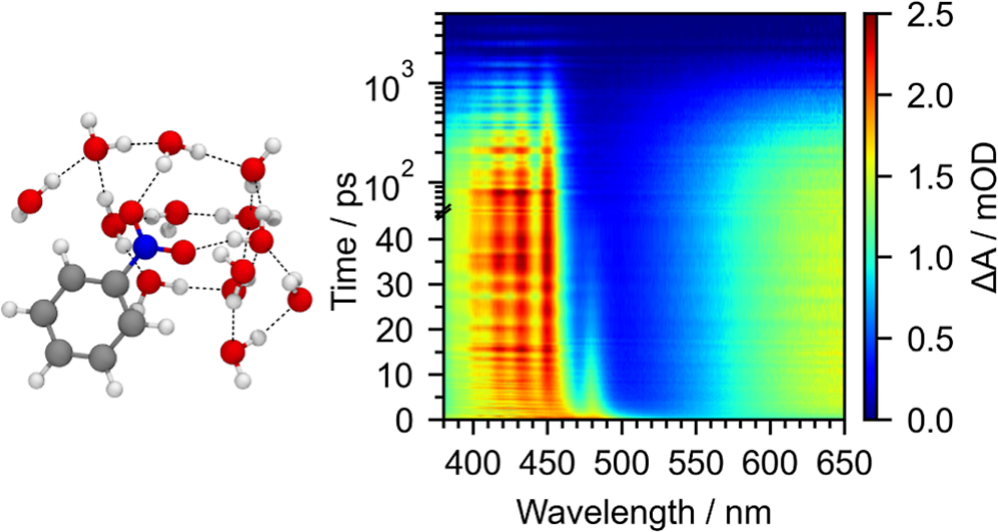

Nitroaromatic compounds are major constituents of the brown carbon aerosol particles in the troposphere that absorb near-ultraviolet (UV) and visible solar radiation and have a profound effect on the Earth’s climate. The primary sources of brown carbon include biomass burning, forest fires, and residential burning of biofuels, and an important secondary source is photochemistry in aqueous cloud and fog droplets. Nitrobenzene is the smallest nitroaromatic molecule and a model for the photochemical behavior of larger nitroaromatic compounds. Despite the obvious importance of its droplet photochemistry to the atmospheric environment, there have not been any detailed studies of the ultrafast photochemical dynamics of nitrobenzene in aqueous solution. Here, we combine femtosecond transient absorption spectroscopy, time-resolved infrared spectroscopy, and quantum chemistry calculations to investigate the primary steps following the near-UV (λ ≥ 340 nm) photoexcitation of aqueous nitrobenzene. To understand the role of the surrounding water molecules in the photochemical dynamics of nitrobenzene, we compare the results of these investigations with analogous measurements in solutions of methanol, acetonitrile, and cyclohexane. We find that vibrational energy transfer to the aqueous environment quenches internal excitation, and therefore, unlike the gas phase, we do not observe any evidence for formation of photoproducts on timescales up to 500 ns. We also find that hydrogen bonding between nitrobenzene and surrounding water molecules slows the S_1_/S_0_ internal conversion process.

## Introduction

Nitroaromatic compounds are abundant in the Earth’s atmosphere, with both natural and anthropogenic sources. They are formed in atmospheric photochemical reactions and by incomplete combustion of wood and fossil fuels.^1−3^ Solar ultraviolet (UV) radiation can photodissociate gaseous nitroaromatic compounds to form noxious NO_*x*_ species, which cause respiratory and cardiovascular diseases^4^ and are photochemical sources of ozone in the troposphere.^5−7^ Nitroaromatic compounds such as nitrophenols and nitrocatechols are also organic constituents of brown carbon (BrC) aerosol particles formed by biomass burning in wildfires, land clearance by deforestation, and agricultural practices.^8−13^ The nitroaromatic chromophores contribute significantly to the absorption of solar near-UV and visible radiation by BrC aerosols^14^ and hence to radiative forcing in the troposphere.^15^

Nitrobenzene is the smallest nitroaromatic molecule and is a model for the tropospherically important chromophores of nitrophenol, nitrocatechol, nitrosyringol, and nitroguaiacol compounds identified in BrC aerosols. It has a broad electronic absorption spectrum spanning UVA (400–315 nm) and UVB (315–280 nm) wavelengths that penetrate the upper atmosphere to reach the troposphere and UVC (280–100 nm) wavelengths that are absorbed by gases such as ozone and oxygen at higher altitudes.^7,16−18^ However, its photophysics and photochemistry are still not understood fully and, perhaps surprisingly, there have been relatively few investigations of photoinitiated processes of nitrobenzene in aqueous solutions,^19−21^ despite water-containing aerosol droplets and particles being ubiquitous in the troposphere.^22^

The absorption spectrum of nitrobenzene has four distinct features in the UVB and UVC regions. In the gas phase, these are centered around 280, 240, 193, and 164 nm.^23^ In addition, a weak absorption feature is also observed in the UVA region around 345 nm.^24^ Computational studies have assigned these features as transitions to singlet excited electronic states.^25−29^ The 345 nm absorption is attributed to a transition to the ^1^(n_A_π*) state, mainly localized on the nitro group and hereafter referred to as S_1_. This transition is optically forbidden at the *C*_2*v*_ symmetry of nitrobenzene in its planar ground-state geometry;^29^ however, the oscillator strength is *f* = 0.02^27^ when the *C*_2*v*_ symmetry is broken by rotation around the C–N bond resulting in an average C_6_H_5_–NO_2_ dihedral angle of around 13°.^29,30^ The 280 nm absorption is attributed to transitions to the ^1^(n_B_π*) and ^1^(L_b_ππ*) states,^28^ hereafter referred to as S_2_ and S_3_, respectively.^29^ Changes in electron density upon excitation to S_2_ are mainly localized on the nitro group and for S_3_ are partly localized on the benzene ring with some charge transfer character from the benzene ring to the nitro group. The 240 nm absorption is attributed to a transition to the ^1^(L_a_ππ*) state, which is a charge transfer state from the benzene ring to the nitro group and hereafter referred to as S_4_.^29^ This band dominates the absorption spectrum since the S_0_–S_4_ oscillator strength is 2 orders of magnitude larger those of S_0_–S_1,2,3_.^29^ The 193 and 164 nm absorptions lie outside the scope of this work and are not considered further. In *n*-heptane, methanol, and water solutions, absorptions have been observed around 340, 250–270, and 200 nm.^23^ The S_0_–S_4_ absorption band shifts to a longer wavelength with increasing solvent polarity,^23^ as expected for a transition to a charge-transfer state.

The photophysics and photochemistry following photoexcitation of nitrobenzene to its S_1_ to S_4_ states have been probed using a range of methods. In the gas phase, the photodissociation of nitrobenzene was first investigated by Galloway et al.^31^ using UV (320–220 nm) and vacuum UV (125 nm) resonance-enhanced multiphoton ionization (REMPI) mass spectrometry. They determined branching ratios for elimination of NO_2_, NO, and O and found that NO_2_ was the dominant photoproduct. They also determined that the NO_2_/NO branching ratio increased with increasing UV photodissociation energy, proposing that the production of both NO_2_ and NO was through a nitrite intermediate at lower photodissociation energies but that direct C–N bond cleavage was possible at higher energies. The same trend was also reported in a more recent study by Lin et al. using multimass ion imaging.^32^ The mechanism for photodissociation was probed in detail by Hause et al. using state-selected direct current slice imaging.^33^ This work revealed two pathways following excitation at 266 nm. One involved an intramolecular rearrangement via an oxaziridine ring in the lowest energy triplet state and the other was attributed to a roaming reaction on the ground electronic state in which NO_2_ and phenoxy products reorient until they isomerize into phenyl nitrite, before eventually decomposing to form NO. Recently, this picture has been challenged by computational CASPT2//CASSCF(14,11) investigations of the photodissociation pathways. This work suggested that the roaming pathway was only accessible following photodissociation at energies ≥5.13 eV (242 nm) and that photodissociation at lower energies occurred via formation of oxaziridine or epoxide rings.^34^ Giussani and Worth have subsequently used the CASPT2//CASSCF method to show that the photodissociation yield can be modified by methyl substitution at the ortho position and by extending conjugation.^35^ Bejoy et al. have also shown that the nature of the ortho substituent can change the branching ratio between the different dissociation pathways, using velocity map imaging following photoexcitation at 266 nm.^36,37^ A recent computational study further investigated the roaming isomerization of nitrobenzene microsolvated by water molecules, with roaming observed despite hydrogen-bond formation.^38^

The first time-resolved measurements for gas-phase nitrobenzene photodissociation were made by He et al. using ultrafast electron diffraction.^39^ Following 267 nm photoexcitation, they observed the formation of NO and phenoxyl radicals on an 8.8 ± 2.2 ps timescale and proposed a mechanism involving a repulsive triplet state following intramolecular rearrangement.^39^ In contrast to the observations of Galloway et al.^31^ and Lin et al.,^32^ they found that NO was the dominant photoproduct. This discrepancy has since been rationalized by Giussani and Worth, who proposed that nitrobenzene undergoes isomerization in an excited state to produce NO directly and that the remaining photodissociation products are produced in the ground state.^29^ There have also been two femtosecond time-resolved photoelectron imaging (TRPEI) studies. Schalk et al. investigated the relaxation dynamics following photoexcitation at 200 nm to a higher-lying ^1^ππ* state.^40^ Timescales of 40 fs and ∼0.5 ps were reported and assigned to ultrafast relaxation from a higher-lying ^1^ππ* state and either internal conversion (IC) to the ground state or release of NO, respectively. More recently, Saalbach et al. reported timescales of ≤30 fs, 160–190 fs, and 90–160 ps, using 267 nm photoexcitation and TRPEI,^41^ which they assigned to IC from S_3_/S_4_ to S_1_, subsequent intersystem crossing (ISC) to the triplet manifold or competing IC to S_0_, and an additional ISC from the triplet manifold to S_0_, respectively. Such ultrafast multiplicity changes in the gas phase have also been reported for benzene^42,43^ and for other nitroarenes in solution.^44−47^ Most recently, Hegazy et al. reported a time scale of 160 fs for ground state recovery, using 267 nm photoexcitation and mega-electron-volt ultrafast electron diffraction, and did not observe any evidence of photofragmentation within the first 5 ps.^48^

In solution, the photodynamics of nitrobenzene were first investigated by Hurley and Testa using electronic energy transfer (EET).^49^ Following photoexcitation in isopropyl alcohol at 336 nm, they observed a remarkably high triplet yield of > 0.6. The first time-resolved measurements were made by Yip et al. using picosecond transient absorption spectroscopy (TAS).^50^ Following photoexcitation at 355 nm in tetrahydrofuran (THF), they extracted dynamical timescales of <5 ps and 770 ± 90 ps, which they attributed to the growth and decay of the triplet-state population. A subsequent picosecond time-resolved transient grating study of nitrobenzene photoexcited at 320 nm in a range of organic solvents and aqueous sodium dodecyl sulfate solution, by Takezaki et al., gave values for the triplet quantum yield of ∼0.8 and time scales of ≤50 ps and ∼0.4–1 ns, which were assigned to the lifetimes of S_1_ and T_1_, respectively.^51^ A more sophisticated time-resolved population grating study of nitrobenzene in ethanol refined the picosecond time scale to 6 ps and revealed an ultrafast time scale of 100 fs, which was assigned to vibrational relaxation within S_1_.^52^ A recent femtosecond TAS study by Crane et al. has reported multiple timescales for relaxation of 267 nm photoexcited nitrobenzene in hexane and isopropanol.^53^ All previously reported time constants and assignments are summarized in Table 1.

**Table 1:** Relaxation Timescales (τ_1_, τ_2_, and τ_3_) Deduced Following Photoexcitation of Nitrobenzene in a Range of Environments at Specified Wavelengths (λ) Using EET, TAS, Transient Grating (TG), Population Grating (PG), Ultrafast Electron Diffraction (UED), and Photoelectron Spectroscopy (PES)^a^

λ/nm	environment	τ_1_/ps	τ_2_/ps	τ_3_/ps	method
366	benzene			∼1000 (T_1_)	EET^49^
355	THF		≤5 (S_1_)	770 ± 90 (T_1_)	TAS^50^
355	ethanol		≤50 (S_1_*→T_1_)	480 ± 50 (T_1_)	TG^51^
355	benzene		≤50 (S_1_*→T_1_)	750 ± 50 (T_1_)	TG^51^
355	heptane		≤50 (S_1_*→T_1_)	400 ± 50 (T_1_)	TG^51^
355	decane		≤50 (S_1_*→T_1_)	600 ± 50 (T_1_)	TG^51^
355	tetradecane		≤50 (S_1_*→T_1_)	900 ± 50 (T_1_)	TG^51^
355	H_2_O/SDS		≤10 (S_1_*→T_1_)	400 ± 50 (T_1_)	TG^51^
320	ethanol	0.1 (S_1_*→S_1_)	6 (S_1_)	480 (T_1_)	PG^52^
267	gas phase		8.8 ± 2.2 (NO release)		UED^39^
200	gas phase	0.04 (^1^ππ* → S_1_)	0.48 (S_1_ IC/NO release)		PES^40^
267	gas phase	<0.03 (S_4_/S_3_ → S_1_)	0.16–0.19 (S_1_)	90–160 (T_1_)	PES^41^
267	isopropanol	<0.2 (S_4_/S_3_ → S_1_)	3.5–3.9 (T_2_ → T_1_)	340–375 (T_1_ → S_0_)	TAS^53^
		<0.1 (S_1_ → T_2_ or S_0_)			
267	hexane	<0.2 (S_4_/S_3_ → S_1_)	6.0–6.7 (T_2_ → T_1_)	1300–1400 (T_1_ → S_0_)	TAS^53^
		<0.1 (S_1_ → T_2_ or S_0_)			

a S_1_* represents vibrationally excited S_1_.

Several computational studies have attempted to rationalize the experimental observations for UV photoexcitation of gas-phase nitrobenzene.^25−29,34^ A recent comprehensive CASPT2//CASSCF(14,11) study has characterized the main decay paths taken following photoexcitation of the lowest S_1_ and brightest S_4_ excited states.^29^ The absence of radiative relaxation was explained by the presence of accessible conical intersections (CIs) and singlet–triplet crossing (STC) regions between the S_4_, S_1_, and T_1_^3^(n_A_π*) and ground states, and the high triplet quantum yield was attributed to strong spin–orbit coupling (SOC) between the S_1_ and T_2_^3^(π_O_π*) states. Additional CIs and STC regions were also identified and proposed to account for the photoproducts observed in gas-phase measurements.^18,31−33,39,54−56^

Motivated by the lack of detailed investigations of the photochemical dynamics of nitrobenzene in aqueous solution and the environmental importance of the near-UV photochemistry of nitroaromatic molecules, we have undertaken a combined femtosecond TAS and time-resolved infrared (TRIR) spectroscopy study of nitrobenzene in aqueous solution, supported by quantum chemistry calculations. We compare these results to analogous measurements undertaken in solutions of methanol (a protic polar organic solvent), acetonitrile (an aprotic polar organic solvent), and cyclohexane (a nonpolar organic solvent) to explore the consequences of different solvent–solute interactions.

## Results and Discussion

### Steady-State Absorption Spectra

**Figure 1:**
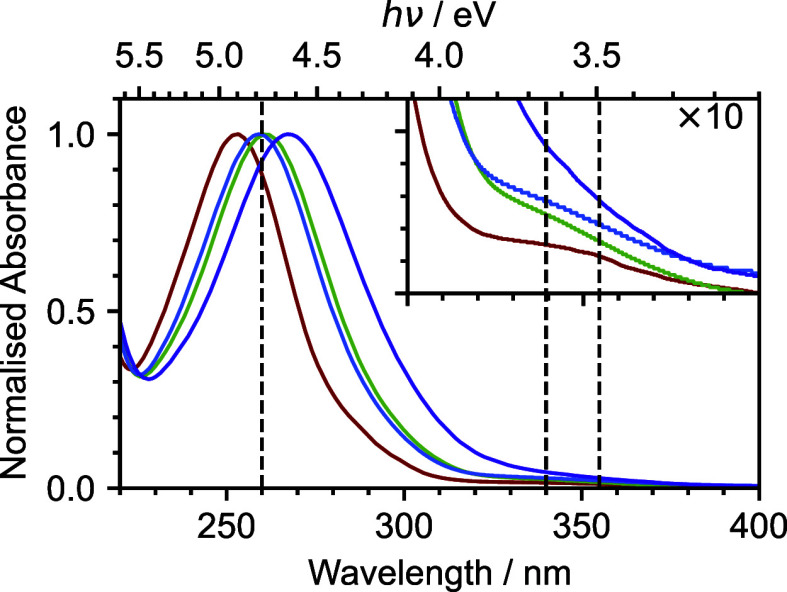
UV–visible spectra of nitrobenzene in water (15 mM, purple), cyclohexane (104 mM, red), acetonitrile (68 mM, green), and methanol (102 mM, blue). Inset: expanded view (×10) of the UV–visible spectra in the 300–400 nm range. The dashed vertical lines mark the wavelengths employed in this work. Data have been normalized to the maximum of the band centered around 250–280 nm.

Steady-state UV–visible absorption spectra recorded in water, cyclohexane, acetonitrile, and methanol are presented in Figure 1 and are consistent with those reported previously.^23,24,41^ The absorption bands around 355 nm (weak), 280–340 nm (stronger), and 250–280 nm (strong) are attributed to S_0_–S_1_, S_0_–S_2_/S_3_, and S_0_–S_4_ transitions, respectively. The spectrum of nitrobenzene in cyclohexane is similar to the gas-phase absorption spectrum,^23^ as expected for a nonpolar solvent in which there are weak solute–solvent interactions. The maximum of the S_4_ band is shifted considerably to longer wavelengths in water (267 nm) compared to cyclohexane (253 nm) as a result of its charge-transfer character. Although the S_1_ shoulder is quite clearly resolved in cyclohexane, it is less distinguishable in water. This is most likely because it is masked by the red-shifted S_4_ band and presumably also the S_3_ band, which has some charge-transfer character. Our femtosecond TAS measurements are recorded following photoexcitation to S_1_ at 355 nm, and our femtosecond TRIR measurements are recorded following photoexcitation to S_1_ at 340 nm and S_4_ at 260 nm.

### Transient Absorption Spectra

**Figure 2:**
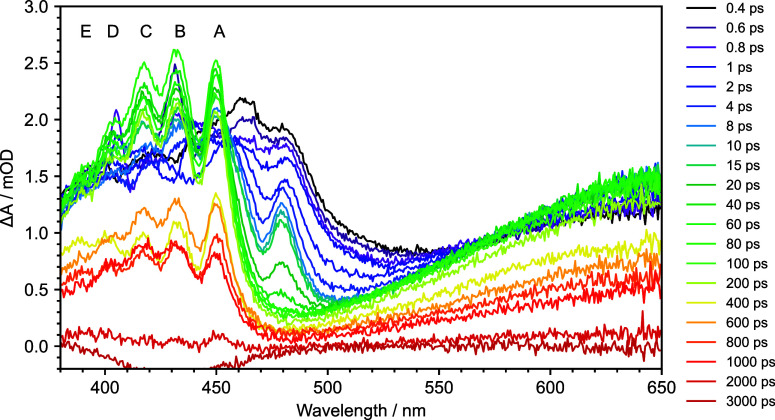
Transient absorption spectra of 15 mM nitrobenzene in water at specified pump–probe delays following photoexcitation at 355 nm. Letters A–E label the peaks in the structured triplet ESA (see text).

Transient absorption maps of nitrobenzene in water (15 mM), cyclohexane (104 mM), acetonitrile (68 mM), and methanol (102 mM), following photoexcitation at 355 nm, are presented in Figures S1–S4. All four maps have similar features. Figure 2 shows a selection of the transient absorption spectra of nitrobenzene in water at pump–probe delays in the range of 400 fs − 3 ns. Immediately on photoexcitation, two absorption features are observed at around 460 and 650 nm. Since 355 nm absorption populates S_1_, we attribute these features to S_1_ excited-state absorptions (ESAs). During the first 10 ps, the broad structureless feature around 460 nm evolves rapidly to reveal a structured ESA and the long-wavelength edge of the S_1_ ESA, at 485 nm, disappears. Until around 80 ps, the center wavelengths of the peaks in the structured ESA gradually shift to shorter wavelengths. At 80 ps, the average peak spacing is 859 cm^–1^ (Table S1). Concomitantly, the feature centered around 650 nm increases in intensity from 1.2 to 1.5 mOD during the first 10 ps, before decreasing in intensity. Nitrobenzene potential energy surfaces from Giussani and Worth^29^ show that only T_1_ and T_2_ lie lower in energy than S_1_, hence the ingrowing structured band around 460 nm and ingrowing band around 650 nm must arise from triplet ESAs following ISC from S_1_. Our observations and assignment are in agreement with those made in the early picosecond TAS study by Yip et al.^50^ and are broadly similar to the recent femtosecond TAS study by Crane et al.^53^

**Figure 3:**
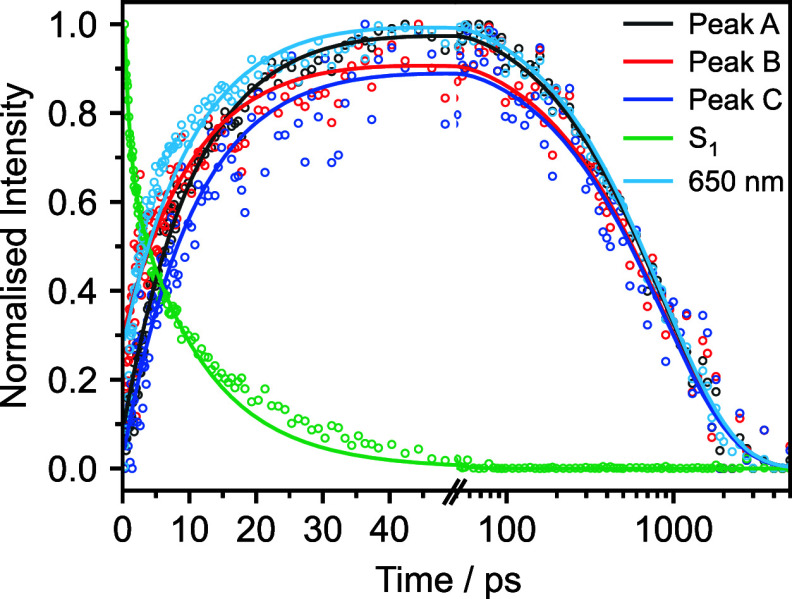
Kinetic traces and associated kinetic fits of the transient absorption spectrum of 15 mM nitrobenzene in water after photoexcitation at 355 nm. Peaks A, B, and C are assigned to the triplet vibrational structure illustrated in Figure 2. S_1_ represents the decay of the ESA from the pump-excited S_1_ state. The broad feature centered around 650 nm is derived from the basis function fitting in the 598–650 nm window. Data have been normalized to the maximum ΔA.

To extract accurate timescales for the relaxation pathways from these overlapping spectral features, it is necessary to decompose the spectra into relevant components. The process of spectral decomposition for the absorption feature centered at 460 nm is presented in Figure S12. The initial population is best represented as a basis function derived from the initial S_1_ ESA. Representation of the in-growing structured triplet feature requires a sum of Lorentzian functions, whose individual Lorentzian components are allowed to shift and narrow to reflect the observed shift to a shorter wavelength and line narrowing. Lorentzian profiles were selected to reflect the dephasing that occurs in liquids.^57^ Shifting and broadening parameters for each transient absorption spectrum are presented in Figures S13–S16. To extract timescales, the scaled amplitude for the broad S_1_ ESA, the amplitudes at the center of each Lorentzian component of the triplet ESA (peaks A, B, and C in Figure 2), and the growth and decay of the broad ESA centered around 650 nm, are plotted against time and fitted with exponential functions convoluted with Gaussian instrument response functions (Figure 3).

The S_1_ decay has two timescales associated with it (0.82 and 11.8 ps, Table 2). The shorter of these is slower than the 100 fs timescale deduced from time-resolved population grating spectroscopy measurements following 320 nm photoexcitation of nitrobenzene in ethanol,^52^ although we note that our corresponding timescale for 355 nm photoexcitation of nitrobenzene in methanol is around 440 fs (Table 2). The slower time scale was fit globally with the growth of peaks A, B, and C, and the feature centered around 650 nm. As we have assigned the structured absorption feature to a triplet ESA, this timescale corresponds to ISC from S_1_ and is consistent with previous transient absorption, transient grating, and population grating spectroscopy studies which reported timescales for ISC from S_1_ ranging from 5 ps to <50 ps.^50−52^ A further 804 ps time constant (Table 2) is associated with the subsequent decay of the triplet excited state and is consistent with timescales reported in previous solution-phase measurements (480–900 ps).^51−53^ We also obtained kinetic traces by plotting the integrated peak areas (Figure S11) instead of the peak heights. The time constants obtained (Table S2) are very similar to those obtained by plotting peak heights; however, there is more scatter and slightly poorer agreement between the rise times of peaks A, B, and C, which we attribute to the complexity of fitting data with overlapping ESAs from both singlet and triplet states.

**Table 2:** Timescales (in ps) Obtained from Fits to the Kinetic Traces (Figures 3 and S8–S10) Following Nitrobenzene Photoexcitation at 355 nm^a^

solvent	τ_IRF_	τ_IC_(S_1_/S_0_)	τ_ISC1_(S_1_/T)	τ_ISC2_(T/S_0_)
water	0.24	0.82 ± 0.28 (0.38)	11.8 ± 0.6 (0.62)	804 ± 23
cyclohexane	0.23	0.25 ± 0.15 (0.43)	12.0 ± 0.2 (0.57)	872 ± 13
acetonitrile	0.27	0.56 ± 0.17 (0.36)	13.8 ± 0.2 (0.64)	800 ± 11
methanol	0.24	0.44 ± 0.02 (0.31)	9.7 ± 0.1 (0.69)	763 ± 10

a τ_IRF_ is the instrument response function, τ_IC_ is the timescale for S_1_/S_0_ IC, τ_ISC1_ is the timescale for ISC from S_1_ to the triplet manifold, and τ_ISC2_ is the timescale for ISC from the triplet manifold to S_0_. The numbers in parentheses are the relative amplitudes associated with the two competing S_1_ relaxation processes.

### Time-Resolved Infrared Spectra

**Figure 4:**
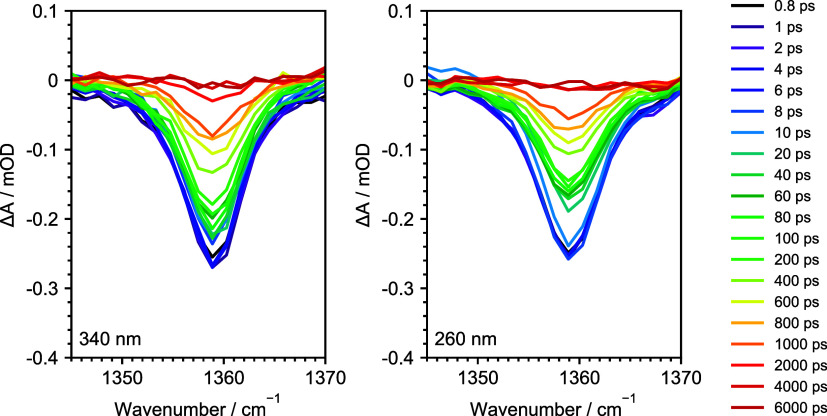
TRIR spectra of 16 mM nitrobenzene in *d*_2_-water at specified pump–probe delays following photoexcitation at 340 nm (left) and 260 nm (right) for the feature centered around 1360 cm^–1^, which is a NO symmetric stretch.^58^

Steady-state Fourier-transform infrared (FTIR) spectra of nitrobenzene in *d*_2_-water (16 mM), cyclohexane (24 mM), and *d*_3_-acetonitrile (16 mM), in the range of 1300–1600 cm^–1^, are presented in Figure S17. Peaks around 1360 and 1530 cm^–1^ are assigned to symmetric and asymmetric NO stretches, respectively.^58^Figure 4 shows TRIR spectra of nitrobenzene in *d*_2_-water at different pump–probe delays after photoexcitation at 340 and 260 nm. Corresponding TRIR spectra of nitrobenzene in cyclohexane and *d*_3_-acetonitrile are presented in Figures S18 and S19. In the TRIR spectra, ground-state bleaches (GSBs) at around 1360 cm^–1^ are observed to decrease to the baseline, indicating complete recovery of the ground state. The baseline remains flat for delays as long as 500 ns, ruling out subsequent formation of photoproducts on the ground electronic state. This is in contrast with 266 nm gas-phase photodissociation measurements, in which NO_2_ and NO were observed on time scales of 13 and 140 ns, respectively,^32^ and polychromatic photolysis measurements, in which a range of aromatic intermediates were observed following irradiation of aqueous nitrobenzene with a 1 kW mercury lamp for 120 min, albeit with low quantum yields.^19^

**Figure 5:**
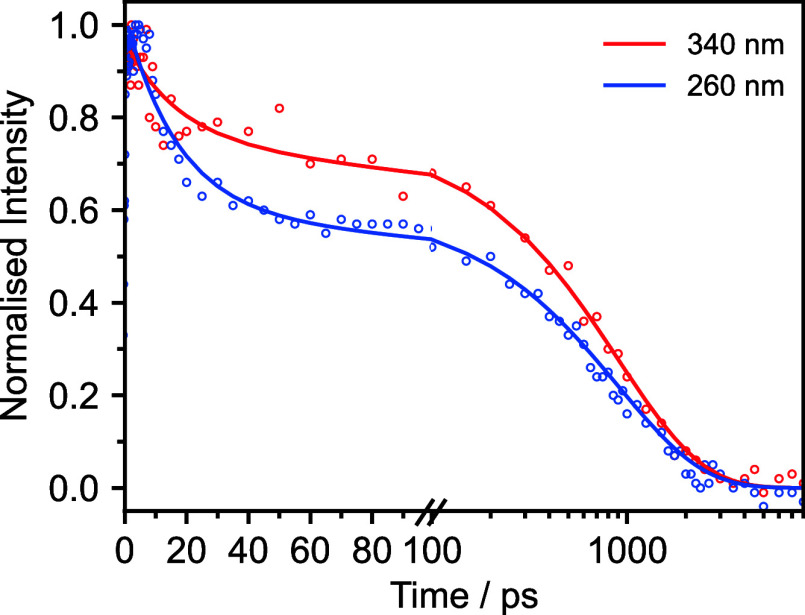
Kinetic traces and associated kinetic fits of the TRIR spectra of 16 mM nitrobenzene in *d*_2_-water after photoexcitation at 340 (red) and 260 nm (blue). Ground-state recovery signals are monitored around 1360 cm^–1^. Data have been normalized to the maximum Δ*A*.

Kinetic traces for the GSB recoveries and associated fits are presented in Figure 5. There are two timescales associated with the recovery of the ground state, one of which is a few tens of picoseconds and the other is around 1 ns (Table 3). The longer timescales are similar to those obtained from our TAS measurements that were assigned to ISC from the triplet manifold to S_0_ (Table 2). However, the shorter timescales (17–30 ps) are up to almost 2 orders of magnitude longer than the TAS timescales that were assigned to S_1_–S_0_ IC (0.4–0.8 ps). This difference can be attributed to the time taken to vibrationally cool in S_0_.^59^ Interestingly, the fast component of the ground-state recovery in *d*_2_-water (∼20 ps) is faster than that in cyclohexane or *d*_3_-acetonitrile (∼30 ps). This comparison is discussed in more detail below.

**Table 3:** Time Constants (in ps) Obtained from Global Fits to the TRIR Spectra Following Photoexcitation at 340 and 260 nm (Figures 4, S18, and S19)^a^

solvent	fitting data (nm)	τ_VR_(S_0_)	τ_ISC2_(T/S_0_)	*A*_VR_	*A*_ISC2_
*d*_2_-water	340	17.1 ± 2.5	900 ± 39	0.22	0.78
	260			0.42	0.58
cyclohexane	340	29.0 ± 5.2	1185 ± 65	0.19	0.81
	260			0.56	0.44
*d*_3_-acetonitrile	340	27.6 ± 2.1	900 ± 28	0.27	0.73
	260			0.69	0.31

a τ_VR_(S_0_) is the time scale for vibrational relaxation in S_0_ following S_1_/S_0_ IC, and τ_ISC2_ is the time scale for ISC from the triplet manifold to S_0_. *A*_VR_ and *A*_ISC2_ are the relative amplitudes associated with these ground-state recovery processes.

### Computational Chemistry Calculations

To support our interpretation of the ESAs observed in our transient absorption measurements, CASPT2(14,11) vertical excitation energies (VEEs) from some of the key geometries on the potential energy landscape^29^ were calculated, both in the gas phase and in aqueous solution, using a polarizable continuum model (PCM). The full set of results and state characterizations are presented in Tables S4 and S5, and the results relevant to interpretation of the TAS measurements are presented in Table 4. Nitrobenzene has a large dipole moment in the ground state, with a calculated value of 4.32 D that compares well with the experimental value of 4.22 D.^60^ The excited singlet states have similar dipole moments and are stabilized relative to S_0_ in an aqueous environment.

**Table 4:** CASPT2(14,11)/ANO-S Calculated VEEs and Corresponding Wavelengths and CASSCF Gas-Phase Oscillator Strengths (*f*) for Selected Vertical Excitations from S_0_, S_1_(n_A_π*), and T_2_(π_O_π*) Minima^a^

		Gas phase			Aqueous solution (PCM)	
transition	structure	VEE/eV	λ/nm	*f*	VEE/eV	λ/nm
S_0_–S_1_	^1^(gs)_min_	3.32	374	0.000002	3.47	357
S_1_–S_2_	(n_A_π*)min1	1.54	807	0.0046	1.72	718
S_1_–S_3_	(n_A_π*)min1	2.01	616	0.0055	1.89	656
S_1_–S_4_	(n_A_π*)min1	2.10	590	0.14	2.48	500
S_1_–S_5_	(n_A_π*)min1	2.81	441	0.11	2.66	466
T_2_-T_4_	(π_O_π*)min3	0.98	1263	0.054	0.88	1400
T_2_-T_5_	(π_O_π*)min3	2.29	540	0.019	2.19	566
T_2_-T_6_	(π_O_π*)min3	2.82	439	0.071	2.78	445
T_2_-T_7_	(π_O_π*)min3	3.21	386	0.027	3.22	384

a VEEs were calculated in the gas phase and in aqueous solution (PCM), using structures optimized at the CASSCF level.

Our TAS measurements (Figure 2) revealed absorption bands centered around 460 and 650 nm immediately on 355 nm photoexcitation to S_1_. Our assignment of these to S_1_ ESAs is consistent with the calculated VEEs from the S_1_^1^(n_A_π*) minimum which has bright S_1_–S_5_ and S_1_–S_3_ transitions at 466 and 656 nm, respectively. The structured absorption band around 460 nm and the band that grows at around 650 nm were attributed to triplet ESAs. From the relaxation pathway proposed by Giussani and Worth,^29^ the system should cross from the ^1^(n_A_π*) state to a region of the triplet manifold where the minimum energy structure has ^3^(π_O_π_min_*) character. The relevant VEEs at the CASPT2 level from this triplet state, optimized in the gas phase and water PCM at the CASSCF level, are listed in Table 4. This state is labeled T_2_(π_O_π*) as it is the T_2_ state at the Franck–Condon point, although T_1_ and T_2_ are nearly degenerated when including the water PCM (Table S5). The triplet ESAs are consistent with the T_2_–T_6_ and T_2_–T_5_ transitions at 445 and 566 nm.

**Figure 6:**
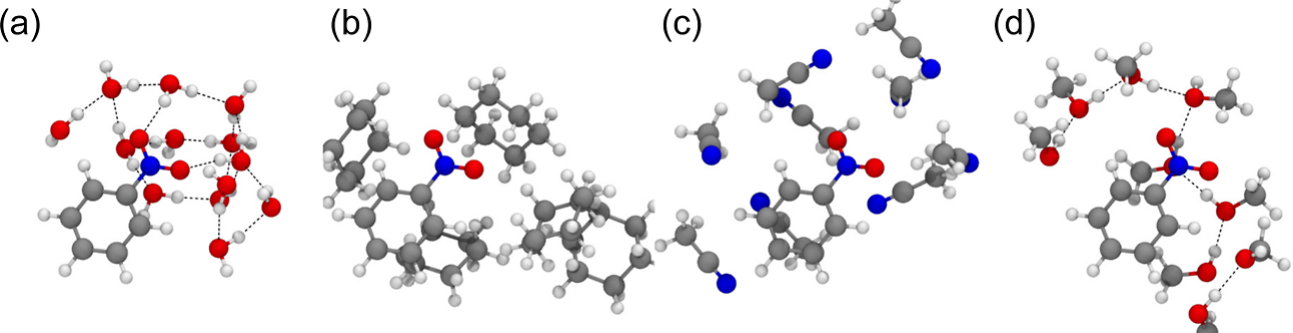
CAM-B3LYP/Def2-SVP GD3BJ microsolvated structures of nitrobenzene using (a) water, (b) cyclohexane, (c) acetonitrile, and (d) methanol. Black dashed lines indicate possible hydrogen bonds.

To inform our interpretation of the slower IC time constant observed in our TAS measurements for nitrobenzene in water compared to cyclohexane, acetonitrile, or methanol, and reported by Crane et al. for hexane and isopropanol,^53^ as well as of the faster ground-state recovery observed in our TRIR measurements for nitrobenzene in *d*_2_-water compared to cyclohexane or *d*_3_-acetonitrile (Table 3), ground-state microsolvation calculations were undertaken (Figure 6). Since none of the solvents are aromatic, the dominant intermolecular interactions are those between the polar nitro group and the solvent. The shortest NO–H intermolecular distances, solvent dipole moments, polarizabilities, and dielectric constants are reported in Table 5. The shortest NO–H intermolecular distances for cyclohexane and acetonitrile microsolvation are around 2.4–2.5 Å, which are indicative of minimal solute–solvent interactions and consistent with the aprotic nature of these solvents. For water microsolvation, the shortest NO–H intermolecular distance is considerably shorter, around 1.9 Å, and there is a hydrogen-bonding network between the NO_2_ group and the immediate water molecules (Figure 6a). Although also protic, the shortest NO–H intermolecular distance in the methanol microsolvated cluster is similar to that in cyclohexane and acetonitrile, and there is no evidence of a hydrogen-bonding network with the NO_2_ group of nitrobenzene, presumably because the higher polarizability of methanol favors dispersion interactions. The insights that emerge from these computational outcomes for the influence of solvent–solute interactions on nitrobenzene photochemistry are discussed below.

**Table 5:** Shortest Intermolecular NO–H Distances, Solvent Dipole Moments, Polarizabilities, and Dielectric Constants for the Solvent Molecules Used in Nitrobenzene Microsolvation Studies^a^

solvent	shortest NO–H intermolecular bond length/Å	dipole moment/D	polarizability/Å^3^	dielectric constant
water	1.90	2.09	6.61	78.4
cyclohexane	2.51	0.00	68.3	2.02
acetonitrile	2.43	4.04	27.0	35.7
methanol	2.48	1.73	18.5	32.6

a Solvated bond lengths were calculated at the CAM-B3LYP/Def2-TZVP GD3BJ level of theory.

### Vibrational Structure in the Triplet ESA Band

The structured absorption band spanning 450–400 nm in the TAS data (Figure 2) is attributed to vibronic transitions associated with the triplet T_2_–T_6_ ESA (Table 4). The peaks shift to shorter wavelengths and narrow with increasing time delay, which can be attributed to vibrational cooling in the triplet manifold,^61,62^ and the band narrowing is dependent on the solvent (Figures S13–S16). The vibrational structure decays uniformly with a time constant of ∼800 ps (Table 2); hence, the structure most likely arises from a vibrational progression in the upper T_6_ state. The average peak spacing in all solvents at a time delay of 80 ps is around 850 cm^–1^. Infrared absorption spectroscopy studies of nitrobenzene in CCl_4_ and the gas phase, and MP2(full)/aug-cc-pVTZ calculations, suggest that ground-state nitrobenzene has an ONO bending vibration with A_1_ symmetry at 850 cm^–1^.^58,63^ It seems likely, therefore, that this is the vibrational mode responsible for the structure in the T_2_–T_6_ ESA. This interpretation is consistent with the recent 266 nm TAS study of nitrobenzene in isopropanol and hexane reported by Crane et al., although we note that their analysis procedure did not allow them to retrieve the band shifting and narrowing evident in our spectra.^53^

### S_1_/S_0_ Internal Conversion

Following photoexcitation to S_1_ at 355 nm, our TAS data reveal S_1_ relaxation times of 0.82 ps in water and <0.6 ps in cyclohexane, acetonitrile, and methanol (Table 2), and our TRIR data (following photoexcitation to S_1_ at 340 nm) reveal that ground-state recovery from direct S_1_/S_0_ IC is 20–30% (Table 3). This IC fraction is consistent with a computational study for gaseous nitrobenzene proposing that 20% of the S_1_ population relaxed back to the ground state via IC^27^ and is supported by the identification of an available conical intersection between S_1_ and S_0_ lying 0.54 eV above the S_1_ minimum.^29^ Following photoexcitation to S_4_ at 260 nm, our TRIR data suggests that >30% of the excited-state population returns to the ground state via IC from S_1_. We can gain some insights into these excitation-wavelength-dependent IC fractions by considering the energies of the key geometries along the relaxation pathways following S_1_ and S_4_ photoexcitation.^29^ In both cases, the crossing point on the S_1_/S_0_ IC seam lies higher in energy than the S_1_/T_2_ STC point; however, the difference in barrier heights between the IC and ISC pathways is slightly lower following photoexcitation to S_4_ compared to that following photoexcitation to S_1_ (0.25 and 0.30 eV, respectively) and could explain why the IC/ISC branching ratio is higher following photoexcitation to S_4_ rather than S_1_.

The S_1_/S_0_ IC timescales also merit discussion. IC is slower for aqueous nitrobenzene than for nitrobenzene in cyclohexane, acetonitrile, or methanol (Table 2). This could be the result of the barrier to the S_1_/S_0_ conical intersection being higher in water because the ^1^(n_A_π*) state is destabilized by the protic solvent. Consequently, excitation with a 355 nm photon would leave less excess internal energy in the nitrobenzene S_1_ molecules when they are photoexcited in water compared to other solvents. Our CASPT2//CASSCF(14,10) calculations confirm this. They show that the minimum energy conical intersection is lower in aqueous solution compared to the gas phase by approximately 0.1 eV but that the S_1_ minimum is lowered much further, by 0.5 eV. Such changes to the S_1_ potential energy surface in aqueous solution could result in it taking longer to reach the S_1_/S_0_ IC from the Franck–Condon region following photoexcitation at 355 nm. Moreover, TAS measurements following photoexcitation at 340 nm reveal a faster S_1_/S_0_ IC timescale of 0.72 ps (Figures S23–S25), compared with 0.82 ps following photoexcitation at 355 nm (Table S3). It is also possible that, since relaxation through the S_1_/S_0_ conical intersection is characterized by activity in the ONO bending mode,^27^ IC could be dynamically hampered by the hydrogen-bonding network between the NO_2_ group and the immediate water molecules described above (Figure 6 and Table 5). After internal conversion to S_0_, vibrational relaxation is faster in water, which is likely a result of stronger coupling between vibrationally hot nitrobenzene molecules to the bath of water molecules (Figure 6 and Table 5).

Regardless of the precise mechanism controlling the rate of S_1_/S_0_ internal conversion in aqueous solution, our observation of separate timescales for S_1_ population relaxation via the S_1_/S_0_ conical intersection and the S_1_/T_2_ STC shows that these nonadiabatic processes are dynamically separated on the S_1_ PES. For example, after photoexcitation to the S_1_ state, there may be a bifurcation of the excited-state dynamics toward these two different crossing points or dynamics that approach the region of the S_1_/S_0_ IC seam before the S_1_/T_2_ STC region. These dynamics must occur in competition with the quenching of excess internal energy in the S_1_ state by coupling to the solvent bath.

### S_1_(*n*_A_π*)/T_2_(π_O_π*) and T_1_(n_A_π*)/S_0_ Intersystem Crossing

Our 355 nm TAS data reveal the timescale of ISC from S_1_ to the triplet manifold to be 11.8 ps for aqueous nitrobenzene and to be largely independent of the solvent (Table 2). We observe similar dynamics following 340 nm photoexcitation (9.9 ps; Table S3). This timescale is slightly longer than those determined in the recent femtosecond TAS study by Crane et al.,^53^ at an excitation wavelength of 267 nm which places greater internal energy into the excited-state nitrobenzene. Our more detailed spectral decomposition of the overlapping ESA bands in the TA spectra of nitrobenzene may also influence the determined timescales.

Our TAS and TRIR data reveal the timescale for subsequent ISC from the triplet manifold back to S_0_ to be around 800–900 ps, again largely independent of the solvent (Tables 2 and 3). Again, the 355 and 340 nm TAS measurements give similar timescales (Table S3). This determination is consistent with the conclusions drawn from transient grating measurements^51^ and supported by computational calculations of the relaxation pathway^29^ that show that efficient ISC occurs from S_1_ to T_2_(π_O_π*) due to the large spin–orbit coupling constant (68 cm^–1^) and the STC being located <0.3 eV higher than the S_1_ minimum. The T_2_(π_O_π*) and T_1_(*n*_A_π*) states are nearly degenerate (Table S5), so while we attribute the triplet ESA to excitation from the T_2_(π_O_π*) state, the IC between the two triplet states is likely to be efficient.

Subsequent T_1_(*n*_A_π*)/S_0_ ISC to repopulate the ground state has been proposed to occur either through an out-of-plane deformation of the NO_2_ group (i.e., distortion along the ONCO out-of-plane improper dihedral angle)^27^ or a planar *C*_2*v*_ geometry.^29^ In the former case, because the STC is 0.15 eV higher than the T_1_ minimum, the geometry must deform from being planar at the T_1_ minimum to an ONCO dihedral angle of around 115° (an out-of-plane dihedral angle of around 65°). At this STC, the spin–orbit coupling was calculated to be 75 cm^–1^.^27^ In the second case, the STC is 0.42 eV higher than the T_1_ minimum and the spin–orbit coupling is also 75 cm^–1^.^29^ If ISC to the ground state requires large structural deformation and some degree of reactivation to reach the STC energy, the relaxation timescale might be expected to depend on the solvent. Our microsolvation calculations indicate that aqueous nitrobenzene might have a longer ISC time constant than in other solvents because the NO_2_ out-of-plane deformation would disrupt the solute–solvent hydrogen-bonding network; however, this solvent dependence is not seen. Therefore, our measurements argue against ISC via NO_2_ out-of-plane deformation, and we instead propose that the population returns to the ground state driven by the coupling from the STC characterized by a planar geometry, as suggested by Giussani and Worth.^29^ Takezaki et al. found the timescale for ISC to S_0_ to increase with decreasing temperature; this is consistent with our interpretation of our experimental observations that the crossing occurs from the T_1_ minimum.^51^

## Conclusions

**Figure 7:**
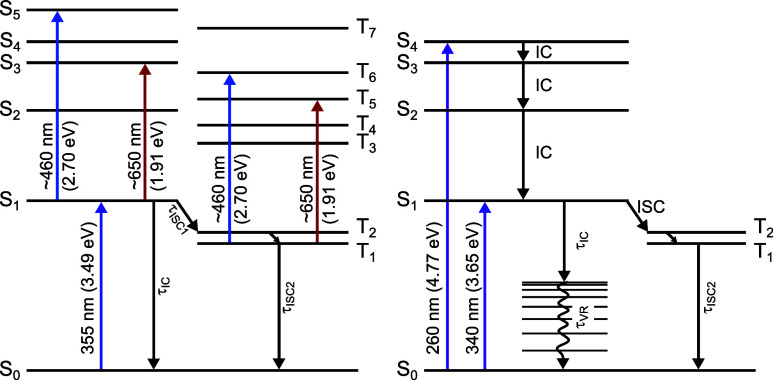
Schematic representation of the relaxation processes following photoexcitation at 355 nm in our TAS experiments (left) and at 340 and 260 nm in our TRIR experiments (right). Note that T_1_ and T_2_ are nearly degenerate.

We report detailed femtosecond to nanosecond TAS and TRIR spectroscopy studies of UV-photoexcited nitrobenzene in aqueous solution, supported by quantum chemistry calculations, and compare the results with analogous measurements for nitrobenzene in cyclohexane, acetonitrile, and methanol. The relaxation pathways and our spectroscopic observations are summarized in Figure 7.

The nonadiabatic pathways for photoexcited nitrobenzene in aqueous solution are broadly similar to those observed in the gas phase^39−41^ and in other solvents.^49−53^ The key differences are that, unlike in the gas phase, we do not observe any evidence for the formation of photoproducts on timescales up to 500 ns because vibrational energy transfer to the solvent rapidly quenches internal excitation in the S_1_ or triplet states, and the hydrogen-bonding interactions between the nitrobenzene NO_2_ group and surrounding water molecules slow the S_1_/S_0_ internal conversion process.

Immediately on photoexcitation to S_1_, transient absorption bands are observed around 460 and 650 nm, which we attribute to S_1_ excited-state absorptions. The rapid decay of the 460 nm ESA band profile within the first 10 ps reveals a structured absorption band that is assigned to the T_2_–T_6_ ESA with a prominent vibrational progression in T_6_. Decomposition of the transient absorption spectra reveals a biexponential decay of the S_1_ ESAs, attributed to S_1_/S_0_ IC and S_1_/T_2_ ISC (followed by rapid T_2_/T_1_ IC) on timescales of 0.82 and 11.8 ps, respectively. S_1_/S_0_ IC is slower in aqueous solution compared to cyclohexane, acetonitrile, and methanol solutions of nitrobenzene, where it occurs on a timescale <0.6 ps. The 11.8 ps timescale for the S_1_ decay matches the growth of the structured ESA band attributed to the population of T_2_. During the next 80 ps, the peak positions in the structured absorption band shift to shorter wavelengths and narrow, as a result of vibrational relaxation in the triplet manifold, before the band intensity decays on a timescale of 804 ps, attributed to T_1_/S_0_ ISC. Complementary TRIR measurements reveal full ground-state recovery occurring on two timescales, 17.1 and 900 ps, attributed to vibrational relaxation in S_0_ following IC from S_1_ and ISC from T_1_, respectively. The IC/ISC branching ratio is found to be around 0.2, following photoexcitation to S_1_. Additional TRIR measurements following 260 nm photoexcitation to S_4_ revealed an increase in the branching ratio. This difference is rationalized in terms of the relative barrier heights of the S_1_/S_0_ conical intersections and S_1_/T_2_ singlet–triplet crossings on the different relaxation pathways.

Nitrobenzene and nitroaromatic molecules containing the nitrobenzene molecular motif play important roles in the atmosphere, both as gaseous molecules and as condensed constituents of aerosol particles. However, their photochemical dynamics in aqueous solutions differ significantly from those in the gas phase. The efficient relaxation pathways of aqueous nitrobenzene photoexcited at UVA and UVB wavelengths suggest that it will be less rapidly photodegraded in aerosol particles than in the gas phase in a lower atmosphere. A detailed understanding of the type presented here for the ultrafast electronic relaxation pathways following near-UV photoexcitation of nitrobenzene in aqueous environments is crucially important for the development of accurate models of nitroaromatic photochemistry in aqueous droplets and organic (e.g., BrC) aerosol particles present in the troposphere.

## Experimental and Computational Methods

### Transient Absorption Spectroscopy

Nitrobenzene (≥99% Sigma-Aldrich), cyclohexane (≥99% Sigma-Aldrich), acetonitrile (≥99.8% Fisher Scientific), and methanol (≥99.8% Fisher Scientific) were purchased and used without further purification. UV–visible absorption spectra of nitrobenzene in deionized water, methanol, acetonitrile, and cyclohexane were recorded using a SHIMADZU UV-3600i Plus spectrophotometer.

Femtosecond TAS experiments have been described previously.^64^ Briefly, femtosecond laser pulses were generated from a regenerative amplifier seeded by a Ti:sapphire oscillator (Coherent Astrella-HE-USP). For UV excitation, tunable laser pulses were generated using an optical parametric amplifier (OPA; Coherent OPerA Solo). Transient absorption spectra were recorded using a commercial transient absorption spectrometer (Ultrafast Systems HELIOS Fire) following photoexcitation at 355 nm (3.49 eV) with pulse energies of 500 nJ at the sample. The broadband probe beam was created via white-light generation by focusing a small portion of the 800 nm fundamental beam into a calcium fluoride plate to give a probing range of 350–650 nm. The relative polarizations of the pump and probe beam were set at the magic angle of 54.7°. Nitrobenzene solutions were flowed continuously at 10 mL min^–1^ through a Harrick flow cell using a liquid diaphragm pump (KNF, SIMDOS 02). The concentrations and path lengths were selected to give an absorbance of around 0.3 at 355 nm: 15 mM water (1000 μm), 104 mM cyclohexane (250 μm), 68 mM acetonitrile (250 μm), and 102 mM methanol (250 μm). NMR studies indicate negligible aggregation in 15 mM aqueous nitrobenzene. The instrument response functions were determined by fitting them to solvent-only spectra.

### Time-Resolved Infrared Spectroscopy

TRIR measurements of UV-photoexcited solutions of nitrobenzene were carried out at the LIFEtime Facility at the STFC Rutherford Appleton Laboratory. This laser system and associated spectrometers have been described in detail previously.^65−69^ In brief, UV excitation laser pulses and mid-IR probe pulses were generated by a synchronized pair of Yb-KGW amplifiers (Light Conversion, PHAROS 100 kHz, 15 W, 260 fs output pulses and PHAROS 100 kHz, 6 W, 180 fs output pulses) seeded by a common Yb:KGW ultrafast oscillator and pumping three OPAs (Light Conversion, ORPHEUS). The output of one OPA was converted to the UV region using second harmonic and sum frequency generation, whereas two OPAs were fitted with difference frequency generation (DFG) units to produce mid-IR probe pulses of bandwidth ∼200 cm^–1^ and partially overlapping spectral ranges. A 12 ns optical delay stage controlled the timing between the UV excitation and mid-IR probe pulses, prior to their spatial overlap at the center of a Harrick cell containing the sample. A peristaltic pump continuously circulated sample solutions through the Harrick cell, which was fitted with CaF_2_ windows separated by 100 μm spacers. The transmitted mid-IR probe pulses were dispersed onto two separate 128-element MCT detector arrays (Infrared Associates). UV excitation energies were 80 nJ/pulse at 260 nm and 800 nJ/pulse at 340 nm with a focal spot size of 150 μm. TRIR spectra were obtained by comparison of measured absorbances with and without the UV excitation pulses.

### Computational Chemistry Calculations

For the microsolvation calculations, nitrobenzene was optimized on the S_0_ surface using the CAM-B3LYP^70^/Def2-TZVP^71^ method employing empirical dispersion with Becke–Johnson damping (GD3BJ).^72^ The analysis of the vibrational frequencies confirmed this geometry as a minimum. Molecules of each solvent system were positioned by hand around the nitrobenzene molecule to maximize interactions with the NO_2_ group, and the resulting microsolvated system was reoptimized at the CAM-B3LYP/Def2-SVP level of theory. The analysis of the vibrational frequencies again confirmed the resulting microsolvated systems as minima. These calculations were performed using the Gaussian 16 program.^73^

Following our earlier work,^29^ all excited-state calculations on the various gas-phase nitrobenzene structures were performed at the CASPT2 level, with the same CAS(14,11) active space as before, which includes the π-orbitals on the ring and the lone pairs on the nitro-oxygen atoms.^29^ The basis set was the atomic natural orbital (ANO) of S-type double-ζ with polarization (ANO-S-VDZP).^74^ The geometries were taken from our earlier work^29^ and calculations were performed using the OpenMolcas program 2023.^75^ The CASSCF wave functions were state averaged over 6 singlets and 5 triplets, except for the triplet excitations from the T_1_(n_A_π*) and T_2_(π_O_π*) minima which included 7 triplet states. Solvation effects were included using a water PCM, with the equilibrium charges from the ground state used for nonequilibrium PCM calculations of the excited states. Structure optimizations were all at the CASSCF level.

## Supporting Information Available

Transient absorption heatmaps, spectral profiles, and kinetics for nitrobenzene in cyclohexane, acetonitrile, and methanol; spectral decomposition process and associated center, width, and amplitude parameters for the spectral decomposition of all transient absorption spectra; time-resolved infrared spectra, kinetics, and spectral decomposition of cyclohexane and *d*_3_-acetonitrile spectra; and computational details (PDF)
Spectral decomposition of nitrobenzene in water (MP4)
Spectral decomposition of nitrobenzene in cyclohexane (MP4)
Spectral decomposition of nitrobenzene in acetonitrile (MP4)
Spectral decomposition of nitrobenzene in methanol (MP4)
