# Supplementary material for: Unraveling the Ultrafast Photochemical Dynamics of Nitrobenzene in Aqueous Solution
Source: J Am Chem Soc. 2024 Apr 4;146(15):10407–17. doi: 10.1021/jacs.3c13826 (PMC11027148; doi:10.1021/jacs.3c13826)
Supplement: Supplementary file 1 — ja3c13826_si_001.pdf [file ja3c13826_si_001.pdf]

Supporting Information: Unravelling the ultrafast photochemical dynamics of nitrobenzene in aqueous solution

Nicholas A. Lau,† Deborin Ghosh,‡ Susannah Bourne-Worster,† Rhea Kumar,† William A. Whitaker,‡ Jonas Heitland,† Julia A. Davies,† Gabriel Karras,¶ Ian Clark,¶ Gregory M. Greetham,¶ Graham A. Worth,† Andrew J. Orr-Ewing,‡ and Helen H. Fielding*,†

†*Department of Chemistry, University College London, 20 Gordon Street, London WC1H 0AJ, UK*

‡*School of Chemistry, University of Bristol, Bristol BS8 1TS, UK*

¶*Central Laser Facility, Research Complex at Harwell, STFC Rutherford Appleton Laboratory, Didcot, Oxfordshire, OX11 0QX, UK*

E-mail: h.h.fielding@ucl.ac.uk

Contents

1 Transient Absorption Maps **S-1**

2 Transient Absorption Spectra **S-3**

3 Transient Absorption Kinetics **S-5**

4 Spectral Decomposition **S-8**

5 Centre, Amplitude, and Width Parameters **S-9**

6 Fourier-Transform Infrared Spectra **S-13**

7 Time-Resolved Infrared Spectra **S-14**

8 Time-Resolved Infrared Kinetics **S-16**

8.1 Extraction of Kinetics S-16

8.2 Global Fits S-17

9 340 nm Transient Absorption Spectra **S-19**

10 Computational Details **S-21**

10.1 Optimised Geometries for Microsolvation Calculations S-21

10.2 Excitation Energies for Nitrobenzene in Gas Phase and PCM Water Environment S-28

References **S-30**

1 Transient Absorption Maps

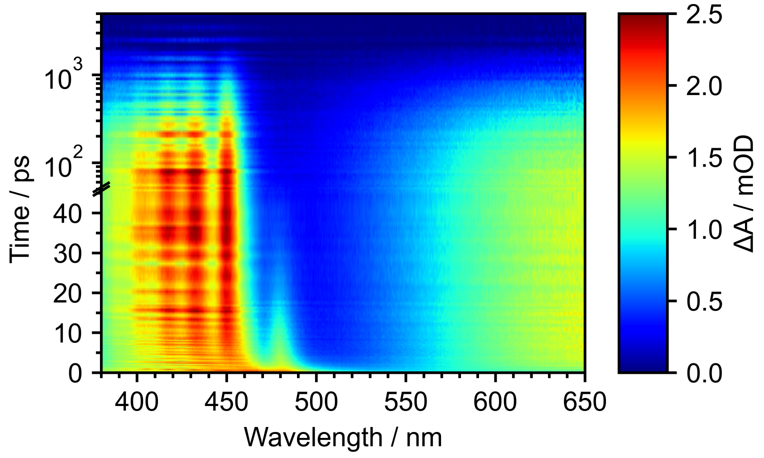

Figure S1: Heatmap of the full 2D transient absorption data for 15 mM nitrobenzene in water following photoexcitation at 355 nm. The time axis has a linear scale from 0-50 ps and a logarithmic scale from 50-5000 ps.

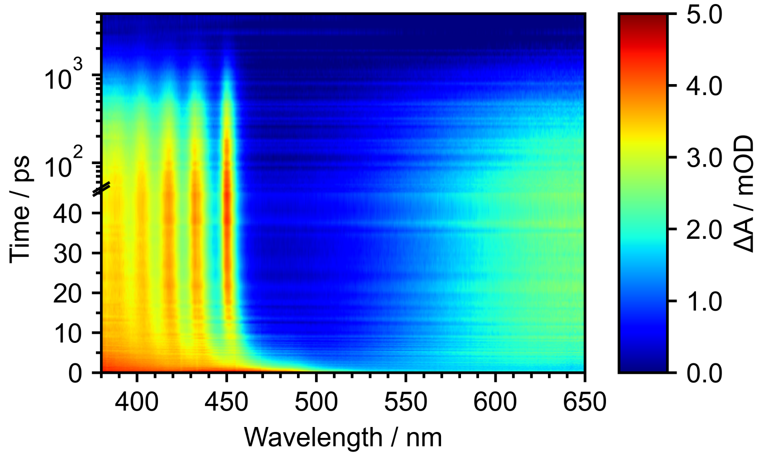

Figure S2: Heatmap of the full 2D transient absorption data for 104 mM nitrobenzene in cyclohexane following photoexcitation at 355 nm. The time axis has a linear scale from 0-50 ps and a logarithmic scale from 50-5000 ps.

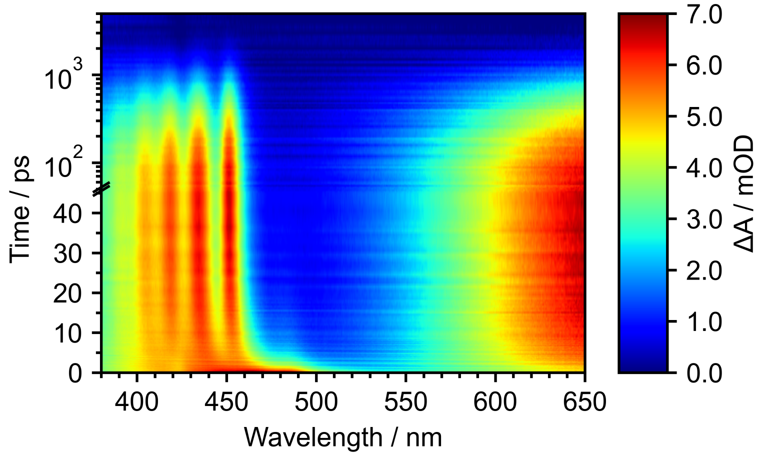

Figure S3: Heatmap of the full 2D transient absorption data for 68 mM nitrobenzene in acetonitrile following photoexcitation at 355 nm. The time axis has a linear scale from 0-50 ps and a logarithmic scale from 50-5000 ps.

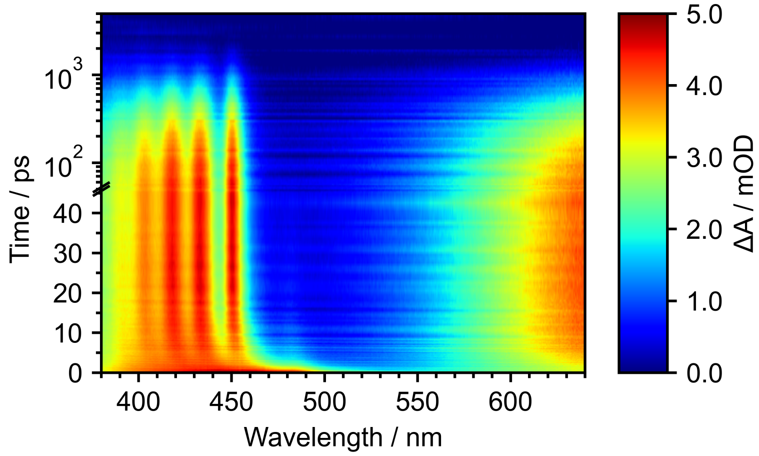

Figure S4: Heatmap of the full 2D transient absorption data for 102 mM nitrobenzene in methanol following photoexcitation at 355 nm. The time axis has a linear scale from 0-50 ps and a logarithmic scale from 50-5000 ps.

2 Transient Absorption Spectra

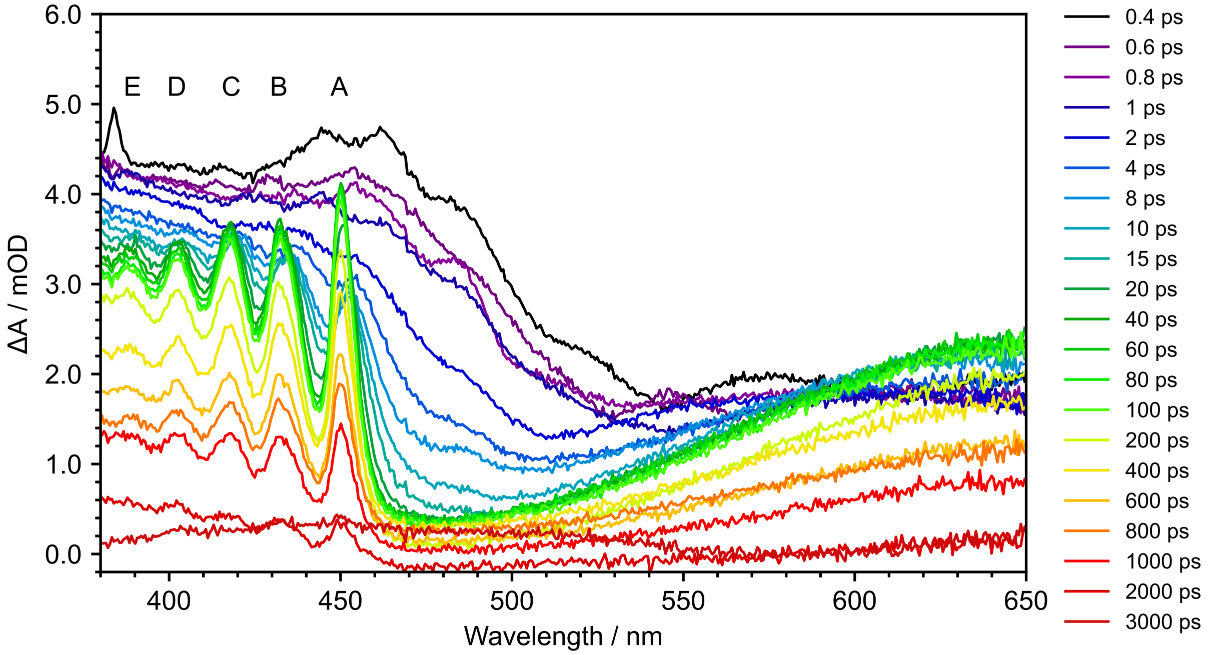

Figure S5: Transient absorption spectra of 104 mM nitrobenzene in cyclohexane displaying pump-probe delays following photoexcitation at 355 nm.

Table S1: Peak spacing for the peaks observed in the structured absorption feature of the transient absorption spectra of nitrobenzene in water (Figure 2 of the main text), cyclohexane (Figure S5), acetonitrile (Figure S6) and methanol (Figure S7). Spacings are determined from the centres of each peak 80 ps after photoexcitation at 355 nm.

	Peak Spacing / cm^{-1}			
Peaks	Water	Cyclohexane	Acetonitrile	Methanol
A-B	976	909	874	879
B-C	733	822	881	820
C-D	781	918	811	881
D-E	947	878	793	760
Average	859	882	840	835

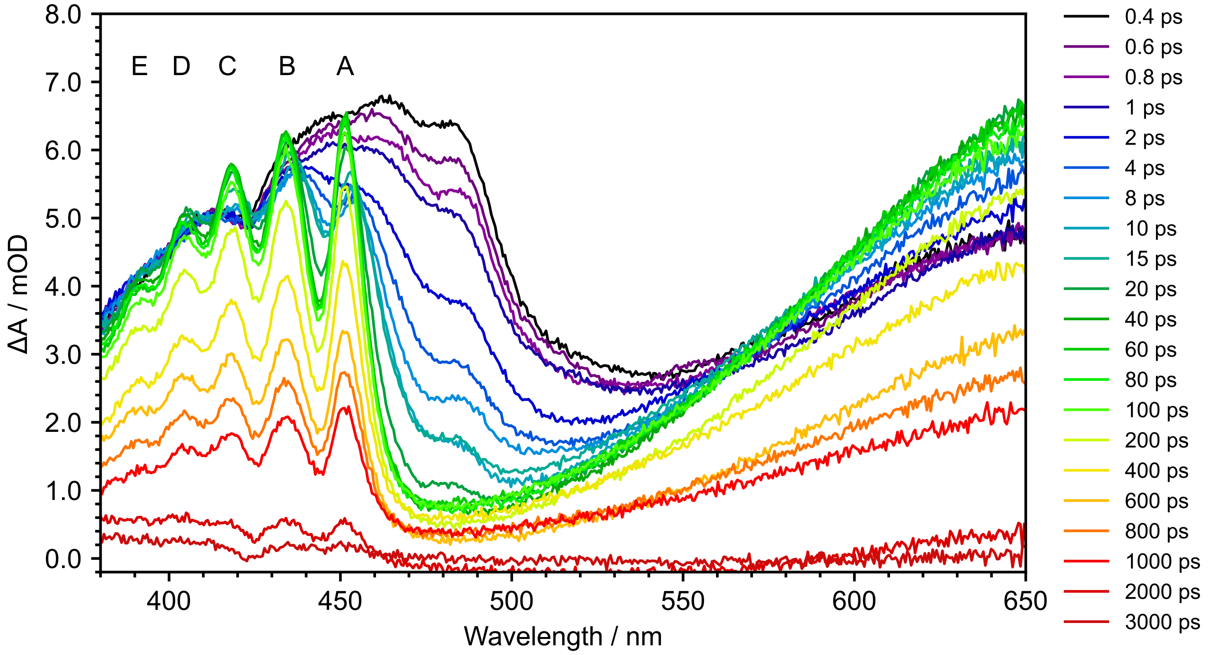

Figure S6: Transient absorption spectra of 68 mM nitrobenzene in acetonitrile displaying pump-probe delays following photoexcitation at 355 nm.

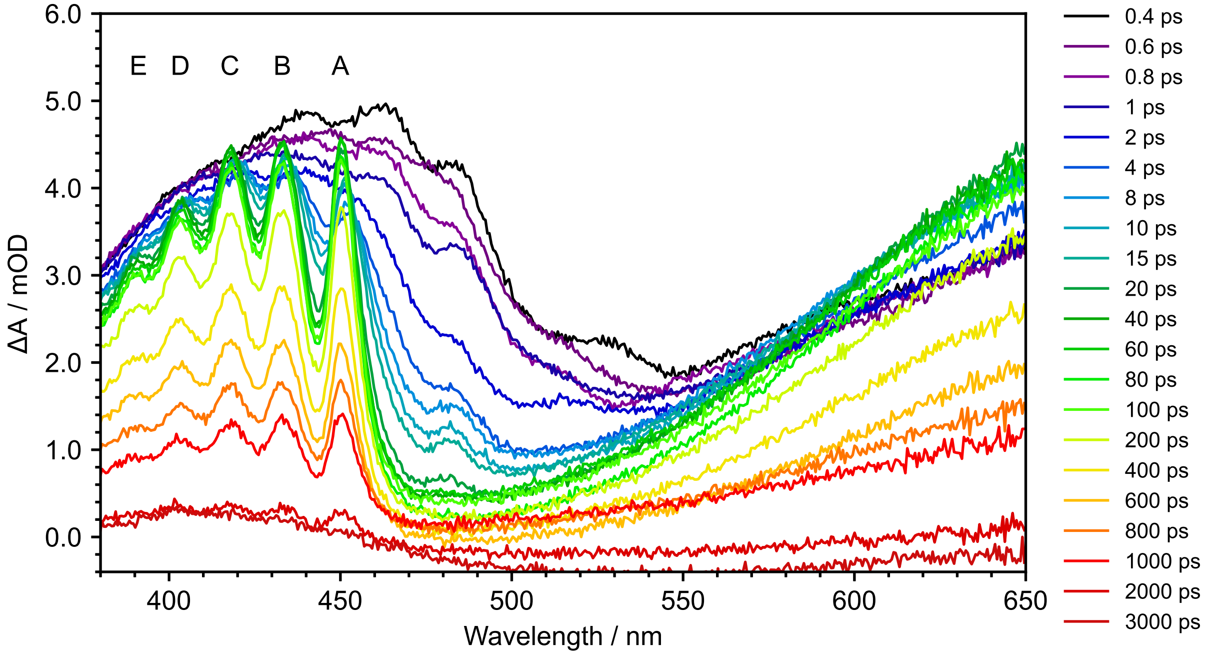

Figure S7: Transient absorption spectra of 102 mM nitrobenzene in methanol displaying pump-probe delays following photoexcitation at 355 nm.

3 Transient Absorption Kinetics

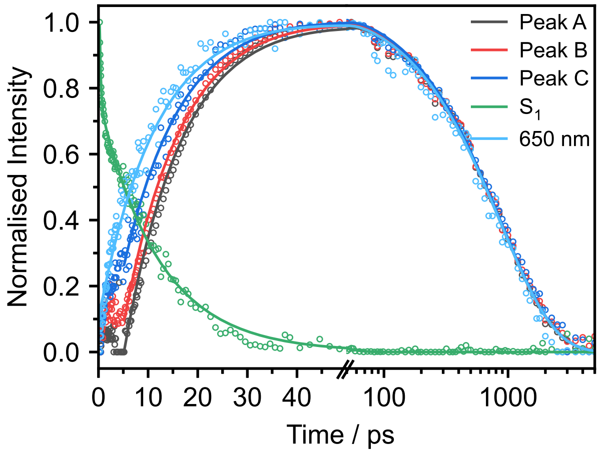

Figure S8: Kinetic traces and associated kinetic fits of the transient absorption spectrum of 104 mM nitrobenzene in cyclohexane after photoexcitation at 355 nm. Peaks A, B, and C are assigned to the T_5 vibrational progression (see Figure 2 of the main text). S_1 represents the decay from the broad pump-excited S_1 state. The broad feature centred around 650 nm is derived from basis function fitting in the 598-650 nm window. Data have been normalised to the maximum ΔA.

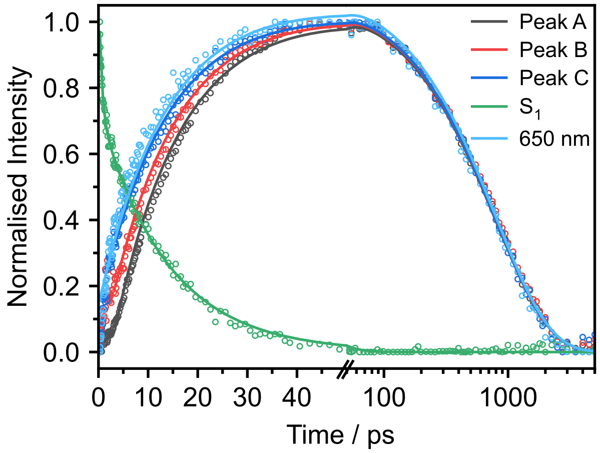

Figure S9: Kinetic traces and associated kinetic fits of the transient absorption spectrum of 68 mM nitrobenzene in acetonitrile after photoexcitation at 355 nm. Peaks A, B, and C are assigned to the T_5 vibrational progression (see Figure 2 of the main text). S_1 represents the decay from the broad pump-excited S_1 state. The broad feature centred around 650 nm is derived from basis function fitting in the 598-650 nm window. Data have been normalised to the maximum ΔA.

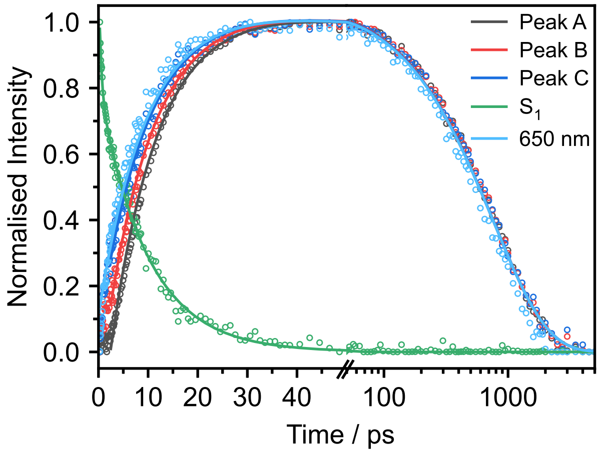

Figure S10: Kinetic traces and associated kinetic fits of the transient absorption spectrum of 102 mM nitrobenzene in methanol after photoexcitation at 355 nm. Peaks A, B, and C are assigned to the T_5 vibrational progression (see Figure 2 of the main text). S_1 represents the decay from the broad pump-excited S_1 state. The broad feature centred around 650 nm is derived from basis function fitting in the 598-640 nm window. Data have been normalised to the maximum ΔA.

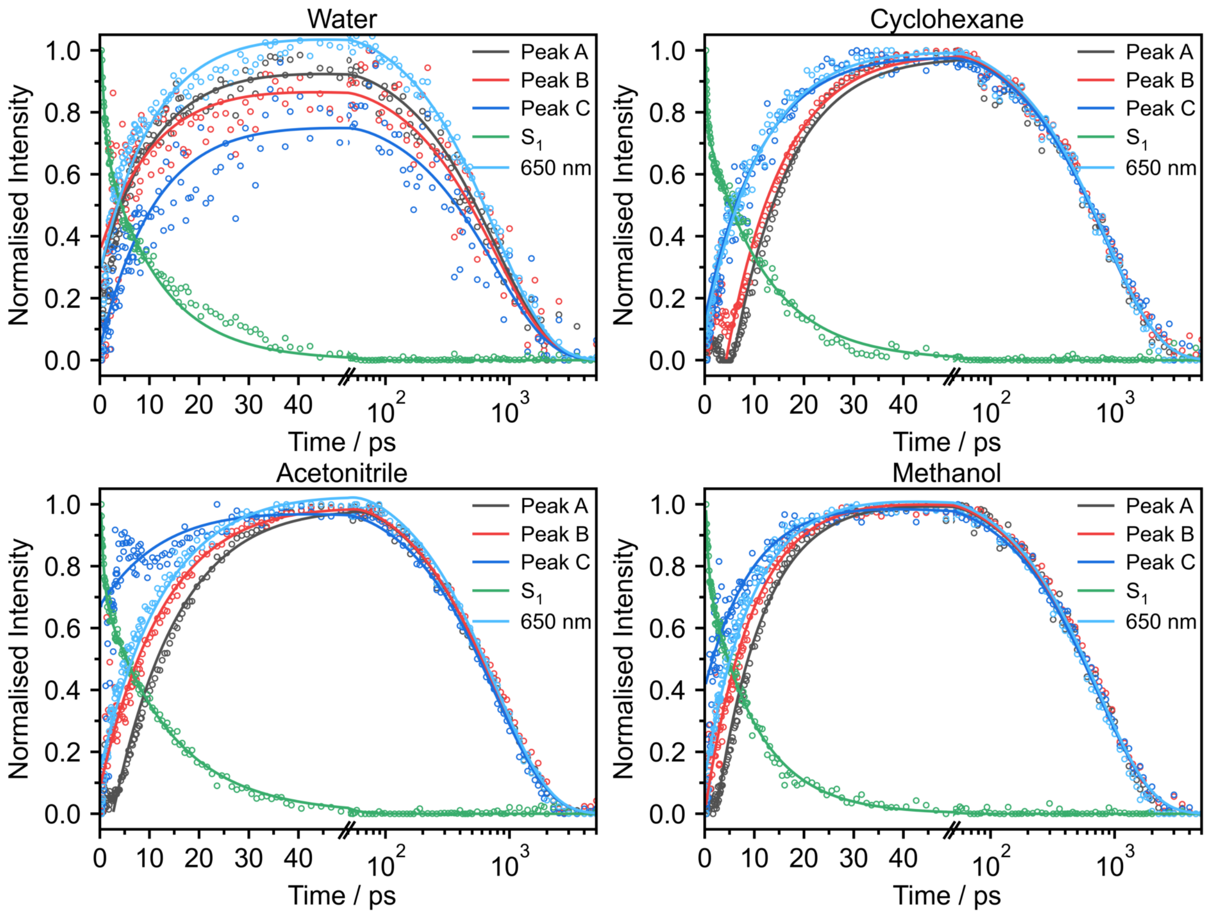

Figure S11: Kinetic traces obtained using integrated areas instead of peak amplitudes.

Table S2: Time constants derived from integrated area fitting.

Solvent	$\tau_{\mathrm{IC}}(\mathrm{S_1/S_0})$	$\tau_{\mathrm{ISC1}}(\mathrm{S_1/T})$	$\tau_{\mathrm{ISC2}}(\mathrm{T/S_0})$
Water	0.76 ± 0.34 (0.38)	11.4 ± 0.8 (0.62)	781 ± 30
Cyclohexane	0.24 ± 0.18 (0.43)	11.9 ± 0.2 (0.57)	856 ± 16
Acetonitrile	0.54 ± 0.23 (0.36)	13.6 ± 0.3 (0.64)	779 ± 15
Methanol	0.49 ± 0.31 (0.31)	9.9 ± 0.2 (0.69)	740 ± 13

4 Spectral Decomposition

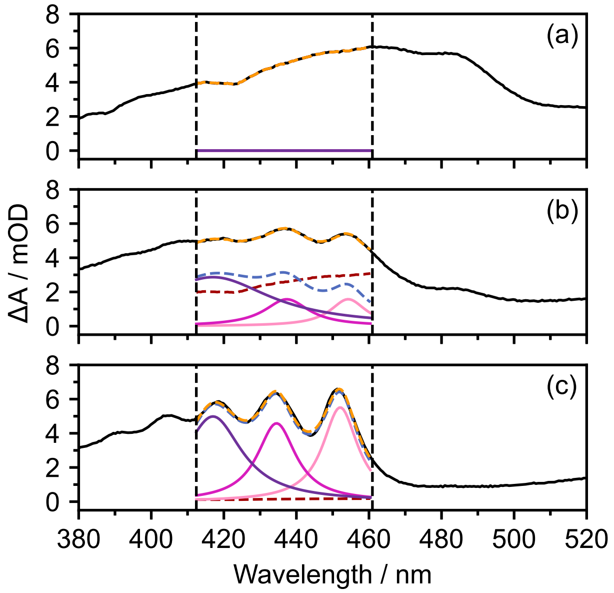

Figure S12: Spectral decomposition of the transient absorption spectrum of 68 mM nitrobenzene in acetonitrile following 355 nm photoexcitation into possible fitting components for the feature centred at 460 nm. The applied fitting window (black dashed lines) covers peaks A, B, and C (see Figure 2 of the main text for peak assignment). The absorption feature is fit using its spectrum as a basis function (red), with the remaining vibrational fine structure fit using a combination of three Lorentzian functions (blue = sum; purple, magenta, and pink = individual Lorentzian fitting functions). Functions are fit to reproduce the original spectrum (orange = reproduction spectrum; black = experimental spectrum). (a) Spectral decomposition of the initial excited state absorption, (b) 6.5 ps, and (c) 42.5 ps.

5 Centre, Amplitude, and Width Parameters

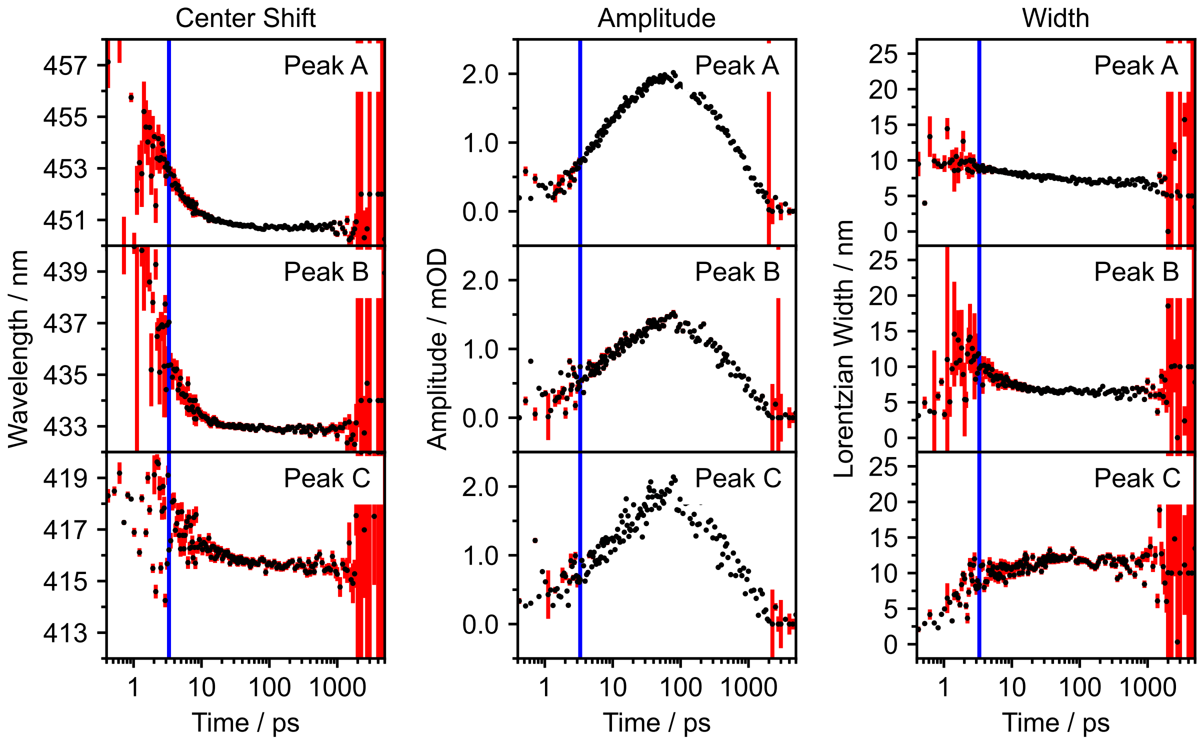

Figure S13: Centre, amplitude, and width parameters extracted from the individual Lorentzian functions used in the spectral decomposition process of the transient absorption spectra for 15 mM nitrobenzene in water. Peaks A, B, and C are assigned to the peaks around 452, 434, and 418 nm respectively (see Figure 2 of the main text). The blue line indicates the position from which kinetic fitting is started. The red lines indicate a one standard deviation error for the parameters derived from the spectral decomposition process.

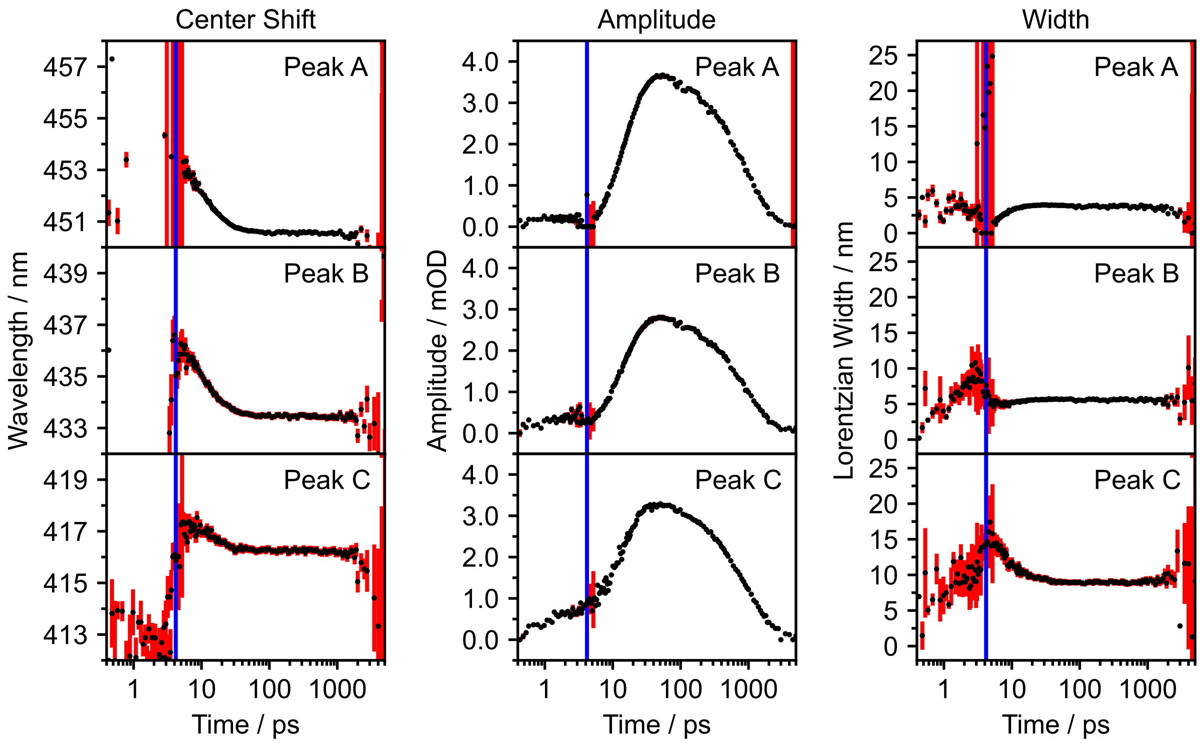

Figure S14: Centre, amplitude, and width parameters extracted from the individual Lorentzian functions used in the spectral decomposition process of the transient absorption spectra for 104 mM nitrobenzene in cyclohexane. Peaks A, B, and C are assigned to the peaks around 452, 434, and 418 nm respectively (see Figure 2 of the main text). The blue line indicates the position from which kinetic fitting is started. The red lines indicate a one standard deviation error for the parameters derived from the spectral decomposition process.

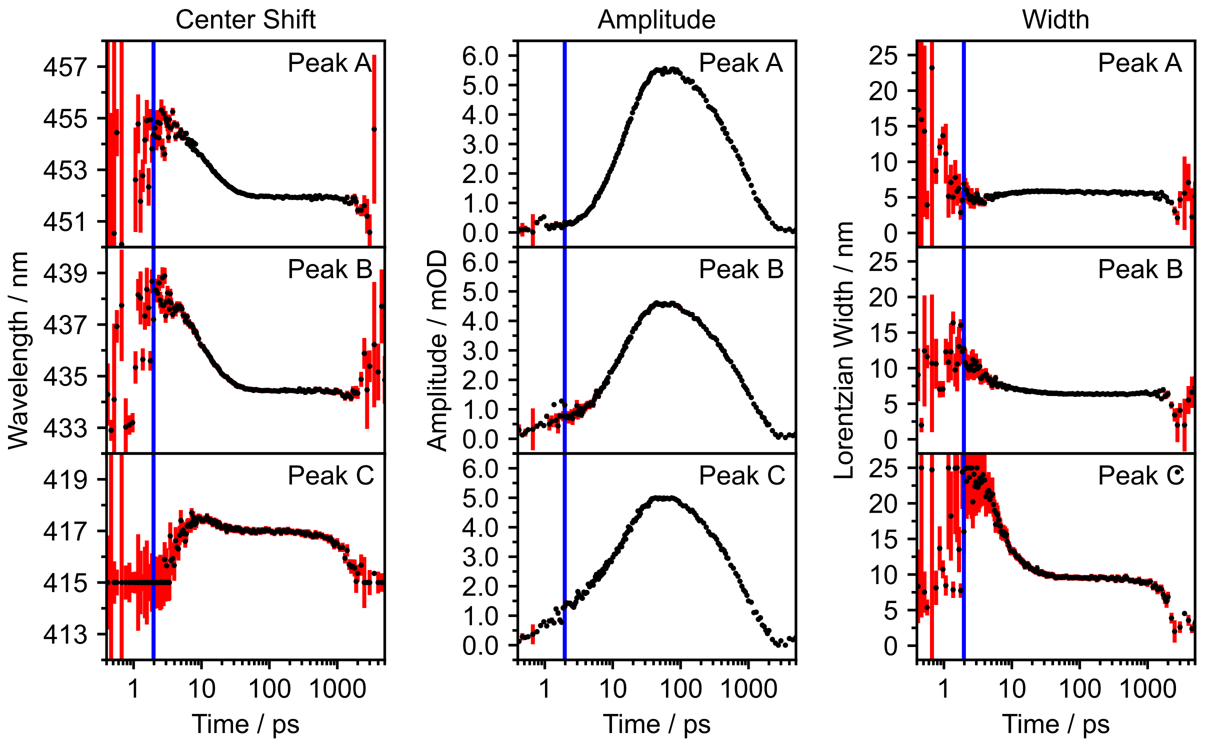

Figure S15: Centre, amplitude, and width parameters extracted from the individual Lorentzian functions used in the spectral decomposition process of the transient absorption spectra for 68 mM nitrobenzene in acetonitrile. Peaks A, B, and C are assigned to the peaks around 452, 434, and 418 nm respectively (see Figure 2 of the main text). The blue line indicates the position from which kinetic fitting is started. The red lines indicate a one standard deviation error for the parameters derived from the spectral decomposition process.

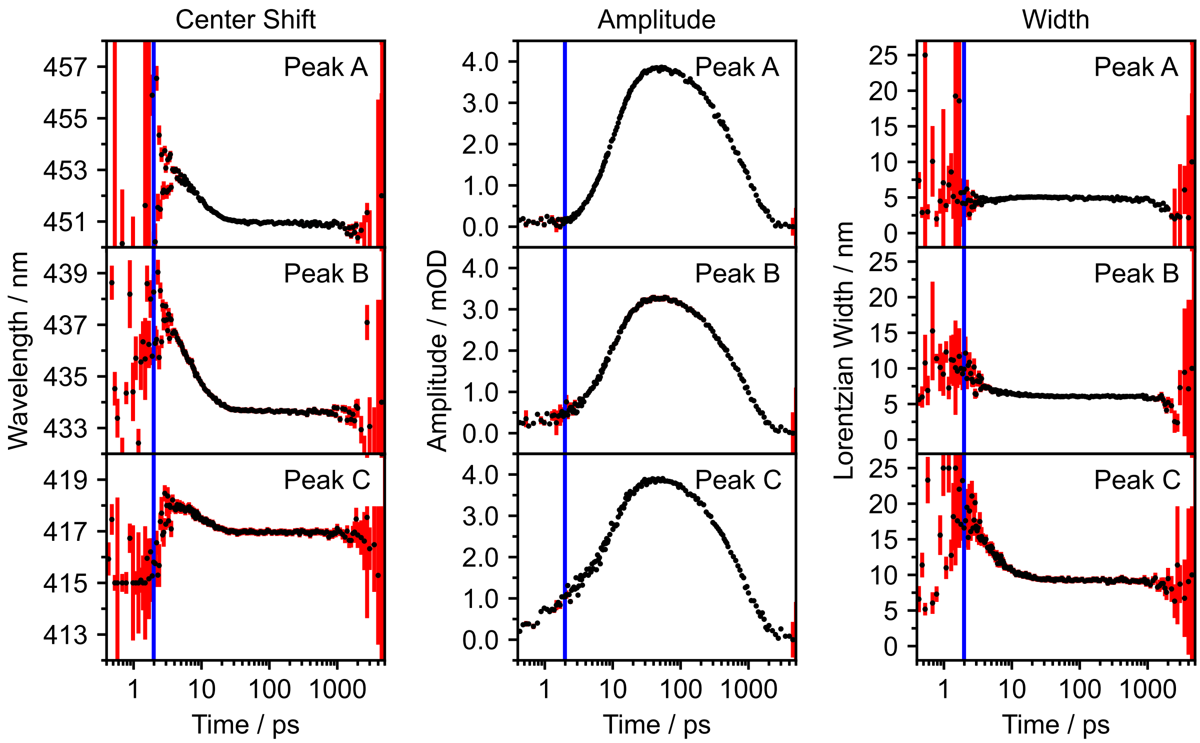

Figure S16: Centre, amplitude, and width parameters extracted from the individual Lorentzian functions used in the spectral decomposition process of the transient absorption spectra for 102 mM nitrobenzene in methanol. Peaks A, B, and C are assigned to the peaks around 452, 434, and 418 nm respectively (see Figure 2 of the main text). The blue line indicates the position from which kinetic fitting is started. The red lines indicate a one standard deviation error for the parameters derived from the spectral decomposition process.

6 Fourier-Transform Infrared Spectra

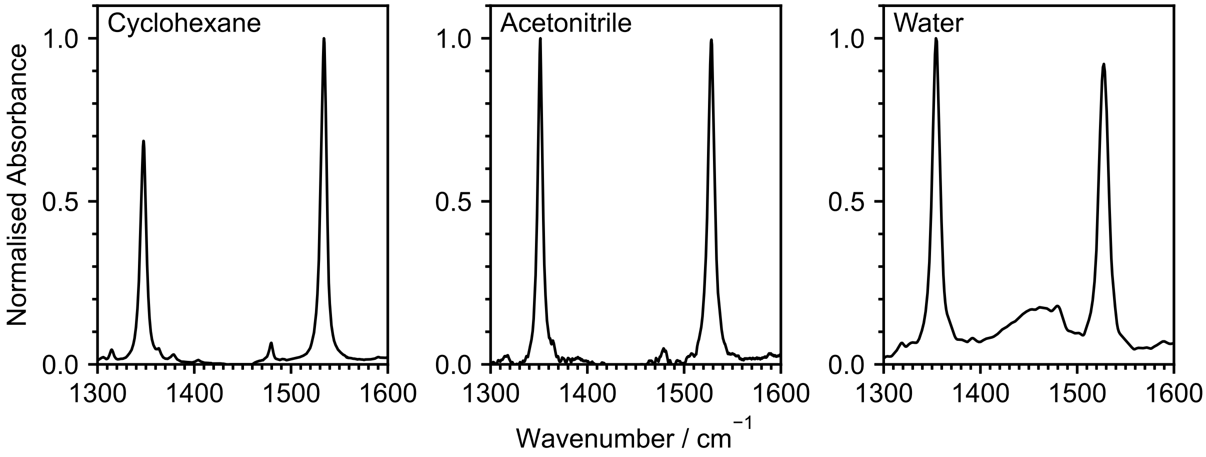

Figure S17: Fourier-transform infrared spectra of nitrobenzene in 24 mM cyclohexane (left), 16 mM d_3-acetonitrile (middle), and 16 mM d_2-water (right).

7 Time-Resolved Infrared Spectra

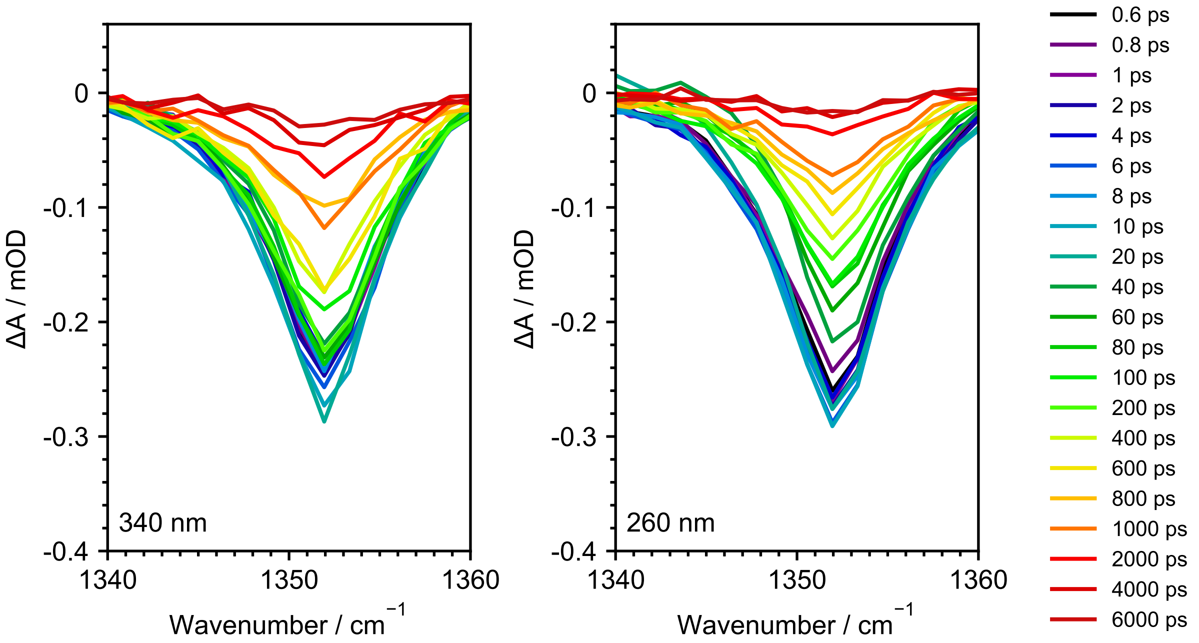

Figure S18: Time-resolved infrared spectra of 24 mM nitrobenzene in cyclohexane displaying pump-probe delays following photoexcitation at 340 nm (left) and 260 nm (right) for the feature centred around 1360 cm^{-1}.

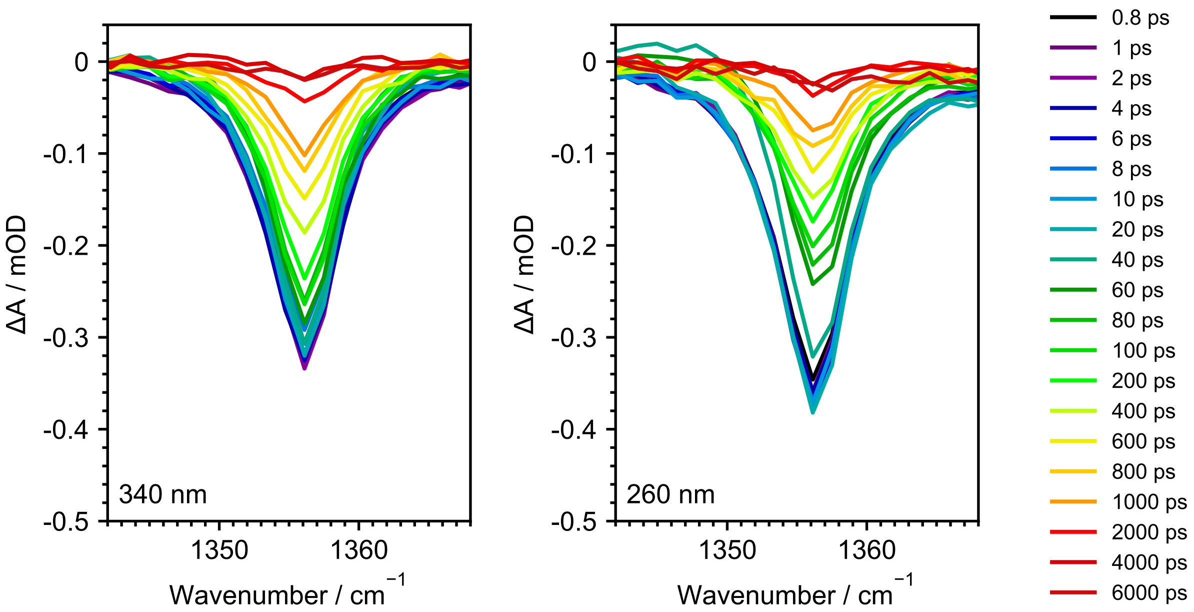

Figure S19: Time-resolved infrared spectra of 16 mM nitrobenzene in d_3-acetonitrile displaying pump-probe delays following photoexcitation at 340 nm (left) and 260 nm (right) for the feature centred around 1360 cm^{-1}.

8 Time-Resolved Infrared Kinetics

8.1 Extraction of Kinetics

Decomposition of all the Time-Resolved Infrared (TRIR) data used the KOALA program.[S1] A Gaussian function was used to fit the ground state bleach (GSB) profile, and a separate Gaussian function with variable central wavenumber fit the hot ground state absorption (HGSA) feature, found to the lower wavenumber side of the GSB, which continuously shifted towards higher energy with increasing time delay. The intensity of the HGSA feature is much less than the GSB, and is only observed in the TRIR spectra following 260 nm photoexcitation. In Figure S20, we present a representative TRIR spectrum to illustrate these Gaussian fits.

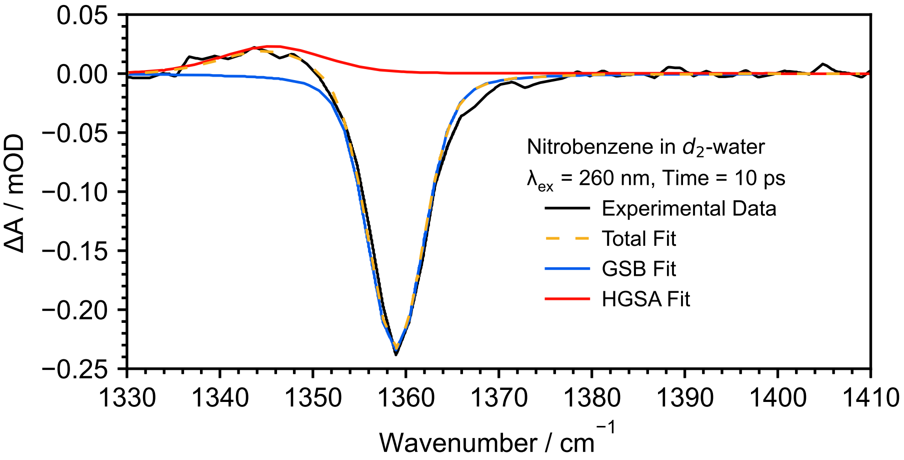

Figure S20: Representative TRIR spectrum of nitrobenzene in d_2-water captured at 10 ps after the 260 nm photoexcitation, with Gaussian fits corresponding to ground state bleach (GSB) and hot ground state absorption (HGSA) spectral features.

The integrated areas of the fitted Gaussian functions describe the band intensities, and changes in these intensities over time reveal the kinetics of HGSA decay and GSB recovery. All TRIR experiments were performed at the STFC Rutherford-Appleton Laboratory (RAL) using the LIFEtime facility.[S2]

8.2 Global Fits

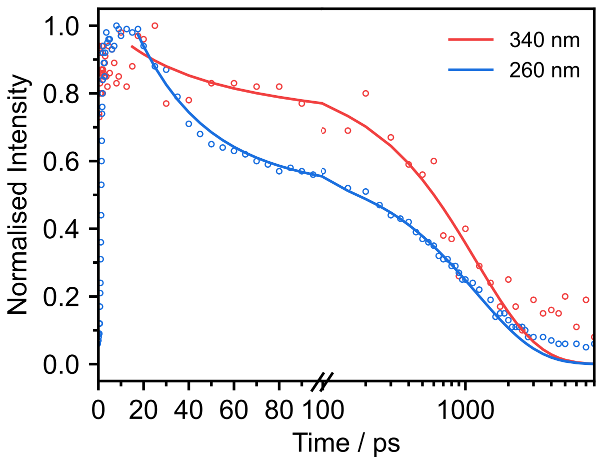

Figure S21: Kinetic traces and associated kinetic fits of the time-resolved infrared spectrum of 24 mM nitrobenzene in cyclohexane after photoexcitation at 340 (red) and 260 nm (blue). Ground state recovery signals are monitored around 1360 cm^{-1}. Data have been normalised to the maximum ΔA.

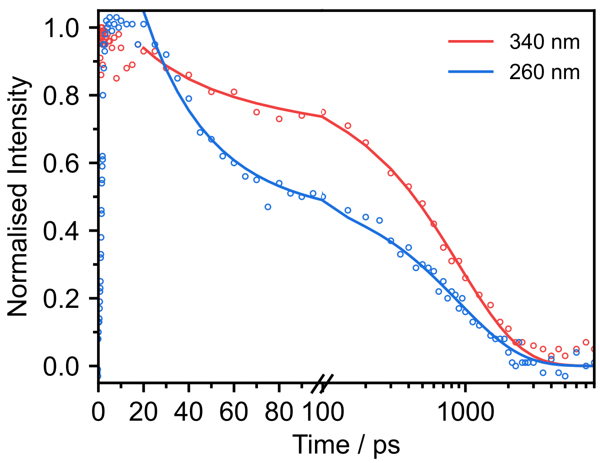

Figure S22: Kinetic traces and associated kinetic fits of the time-resolved infrared spectrum of 16 mM nitrobenzene in d_3-acetonitrile after photoexcitation at 340 (red) and 260 nm (blue). Ground state recovery signals are monitored around 1360 cm^{-1}. Data have been normalised to the maximum ΔA.

9 340 nm Transient Absorption Spectra

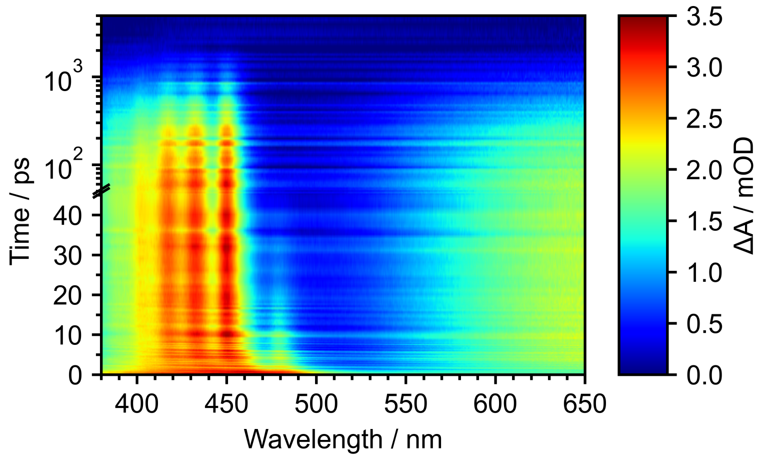

Figure S23: Heatmap of the full 2D transient absorption data for 15 mM nitrobenzene in water following photoexcitation at 340 nm. The time axis has a linear scale from 0-50 ps and a logarithmic scale from 50-5000 ps.

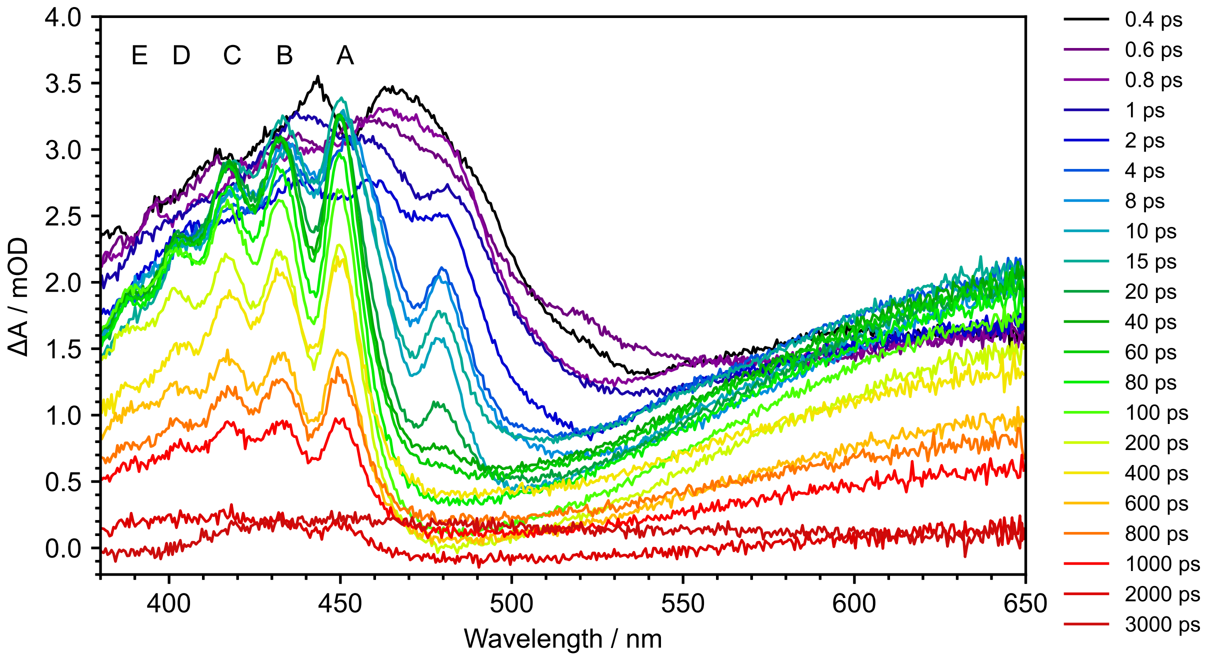

Figure S24: Transient absorption spectra of 15 mM nitrobenzene in water at specified pump-probe delays following photoexcitation at 340 nm. Letters A-E label the peaks in the structured triplet ESA.

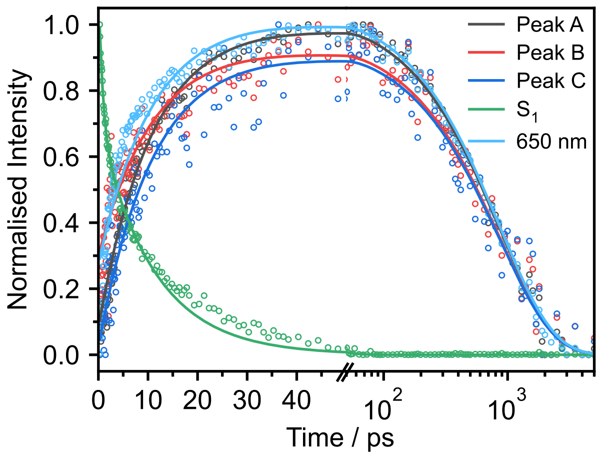

Figure S25: Kinetic traces and associated kinetic fits of the transient absorption spectrum of 15 mM nitrobenzene in water after photoexcitation at 340 nm. Peaks A, B, and C are assigned to the triplet vibrational structure illustrated in Figure S24. S_1 represents the decay of the ESA from the pump-excited S_1 state. The broad feature centred around 650 nm is derived from basis function fitting in the 598-650 nm window. Data have been normalised to the maximum ΔA.

Table S3: Timescales (in ps) obtained from fits to the kinetic traces following nitrobenzene photoexcitation at 340 nm compared with those at 355 nm. τ_{IRF} is the instrument response function, τ_{IC} is the timescale for S_1/S_0 IC, τ_{ISC1} is the timescale for ISC from S_1 to the triplet manifold, and τ_{ISC2} is the timescale for ISC from the triplet manifold to S_0.

λ/nm	τ_{IRF}	$\tau_{IC}(S_1/S_0)$	$\tau_{ISC1}(S_1/T)$	$\tau_{ISC2}(T/S_0)$
340	0.25	0.72 ± 0.18	9.9 ± 0.6	833 ± 24
355	0.24	0.82 ± 0.28	11.8 ± 0.6	804 ± 23

10 Computational Details

10.1 Optimised Geometries for Microsolvation Calculations

All structures optimised at the CAM-B3LYP/Def2-TZVP level of theory.

Nitrobenzene in Water

Atom	x	y	z
C	-3.31599	3.25985	-1.40737
C	-2.22347	3.72532	-0.67676
C	-1.48594	2.85543	0.11869
C	-1.88218	1.52234	0.15693
C	-2.95865	1.02188	-0.57041
C	-3.67779	1.91390	-1.35766
H	-3.88853	3.95242	-2.02819
H	-1.93503	4.77681	-0.72653
H	-0.60415	3.20029	0.66785
H	-3.21864	-0.03885	-0.54225
H	-4.52647	1.54981	-1.93929
N	-1.11926	0.60061	0.99112
O	-1.46638	-0.57078	1.02148
O	-0.18652	1.03162	1.63005
O	0.01534	-2.92357	0.39716
H	-0.39184	-2.11340	0.75095
H	0.89157	-2.96517	0.83101
O	1.29322	4.16038	1.06259
H	1.92096	3.48154	1.37712
H	1.73055	4.50305	0.27381
O	-2.50514	-4.13667	-0.06259
H	-2.85130	-3.85544	0.80722
H	-1.55090	-3.95381	0.01759
O	-4.03130	-2.08097	-0.93294
H	-3.42109	-2.85649	-0.83742
H	-4.71888	-2.39016	-1.53419
O	-3.74428	-2.44357	1.85892
H	-4.11182	-2.15280	1.00200
H	-3.08284	-1.76122	2.03636
O	2.26320	-0.14305	2.53055
H	1.32140	0.03834	2.37617
H	2.43157	-0.99142	2.07641
O	3.25263	2.16297	1.65355
H	2.94651	1.28957	2.02522
H	4.05469	2.37494	2.14588

O	2.68180	-2.65283	1.22014
H	3.13528	-2.61826	0.33815
H	3.20520	-3.26954	1.74724
O	0.99084	-2.02566	-2.08630
H	0.53128	-2.46977	-1.34449
H	1.92387	-2.26883	-1.94107
O	3.72486	-2.50916	-1.27382
H	4.26808	-3.20291	-1.66787
H	4.19051	-1.64648	-1.51525
O	0.63252	0.56800	-1.49820
H	0.03556	0.93754	-2.15972
H	0.75224	-0.37887	-1.77834
O	4.83662	-0.22004	-1.82186
H	4.22035	0.52564	-1.54503
H	5.16578	0.04944	-2.68707
O	3.14818	1.65833	-1.11584
H	2.21963	1.35607	-1.23701
H	3.21088	1.90151	-0.17068

Nitrobenzene in Cyclohexane

Atom	x	y	z
C	-3.15179	3.78929	1.63209
C	-3.94624	2.95112	0.85213
C	-3.41030	2.32764	-0.26821
C	-2.07843	2.56361	-0.58218
C	-1.26577	3.39327	0.17992
C	-1.81624	4.00812	1.29828
H	-3.57649	4.27310	2.51379
H	-4.98900	2.77406	1.12102
H	-3.99583	1.66135	-0.89882
H	-0.22714	3.54108	-0.11096
H	-1.19605	4.66023	1.91558
N	-1.50911	1.90987	-1.77097
O	-0.36118	2.17273	-2.06020
O	-2.21986	1.14614	-2.38768
C	1.60294	-2.10631	-2.48597
C	0.70280	-1.22065	-3.34549
C	-0.66072	-1.86800	-3.58350
C	-1.33602	-2.24954	-2.26716
C	-0.43657	-3.13730	-1.40823
C	0.92424	-2.48504	-1.17143
H	-1.30864	-1.18673	-4.15565
H	0.55404	-0.25111	-2.84218
H	1.19220	-0.99591	-4.30664
H	1.84700	-3.02842	-3.04358

H	2.56077	-1.60261	-2.28564
H	-1.57688	-1.32677	-1.71191
H	-2.29767	-2.74922	-2.46422
H	-0.92408	-3.36130	-0.44570
H	-0.29147	-4.10721	-1.91680
H	0.77873	-1.57667	-0.56335
H	1.57483	-3.15139	-0.58295
H	-0.53081	-2.77661	-4.19888
C	0.51254	-0.15384	2.12737
C	-0.14894	0.87238	3.04555
C	-1.66743	0.70412	3.06522
C	-2.06938	-0.72115	3.44134
C	-1.41010	-1.74262	2.51656
C	0.10787	-1.57875	2.50161
H	-2.12680	1.42907	3.75582
H	0.24407	0.74675	4.07011
H	0.11792	1.89457	2.73343
H	0.21075	0.04781	1.08436
H	1.60700	-0.04482	2.15587
H	-1.75948	-0.92345	4.48202
H	-3.16492	-0.82868	3.41660
H	-1.68361	-2.76729	2.81436
H	-1.79536	-1.60543	1.49118
H	0.50737	-1.81444	3.50413
H	0.56296	-2.30301	1.80992
H	-2.06716	0.93661	2.06368
C	-5.20582	-2.59193	-1.21243
C	-5.35561	-1.07980	-1.37671
C	-5.94623	-0.44279	-0.11861
C	-5.12103	-0.78676	1.12066
C	-4.97298	-2.29859	1.28612
C	-4.38687	-2.94035	0.02947
H	-6.02768	0.65022	-0.23924
H	-4.36351	-0.64005	-1.58066
H	-5.98239	-0.84942	-2.25264
H	-6.20806	-3.04826	-1.12534
H	-4.74268	-3.02816	-2.11110
H	-4.11827	-0.33557	1.02459
H	-5.57496	-0.34341	2.02173
H	-4.34497	-2.52810	2.16085
H	-5.96545	-2.73817	1.49022
H	-3.35416	-2.58109	-0.11011
H	-4.32040	-4.03300	0.15172
H	-6.97838	-0.80959	0.02220
C	4.06003	2.53690	-2.34752

C	3.96959	3.71175	-1.37408
C	2.70587	3.62707	-0.51815
C	2.61936	2.28939	0.21588
C	2.71049	1.11536	-0.75708
C	3.97060	1.19823	-1.61593
H	2.67222	4.46347	0.19903
H	4.85507	3.70398	-0.71353
H	3.99834	4.66707	-1.92189
H	3.23088	2.60550	-3.07327
H	4.99266	2.59362	-2.93102
H	3.44435	2.22517	0.94809
H	1.68727	2.22761	0.79875
H	2.67859	0.16096	-0.21052
H	1.82433	1.12941	-1.41161
H	4.86011	1.07797	-0.97157
H	3.99583	0.36677	-2.33835
H	1.82580	3.73322	-1.17669
C	6.21092	-2.44511	0.28384
C	4.69362	-2.37697	0.11639
C	3.99128	-2.13937	1.45249
C	4.53611	-0.90044	2.16206
C	6.05354	-0.97105	2.32878
C	6.75058	-1.20045	0.98803
H	2.90581	-2.04590	1.29987
H	4.44487	-1.55313	-0.57335
H	4.31635	-3.29805	-0.35542
H	6.47051	-3.33790	0.88040
H	6.69872	-2.57299	-0.69547
H	4.28256	-0.00380	1.57087
H	4.04707	-0.77448	3.14135
H	6.42999	-0.05142	2.80438
H	6.30487	-1.80111	3.01291
H	6.58606	-0.32059	0.34097
H	7.83979	-1.28202	1.13093
H	4.13920	-3.02095	2.10152

Nitrobenzene in Acetonitrile

Atom	x	y	z
C	2.76609	-1.53109	-2.79737
C	2.68327	-0.34590	-2.06969
C	1.51879	-0.02829	-1.38221
C	0.44531	-0.90604	-1.46124
C	0.49876	-2.08979	-2.18624
C	1.67741	-2.40134	-2.85310
H	3.69642	-1.78493	-3.30782

H	3.52483	0.34444	-2.02315
H	1.44359	0.88282	-0.79229
H	-0.37068	-2.74440	-2.20417
H	1.74917	-3.33274	-3.41754
N	-0.79280	-0.56513	-0.75818
O	-1.78742	-1.21604	-1.00909
O	-0.76201	0.35704	0.02848
C	3.74271	2.96620	0.18069
N	3.47384	2.76696	-0.92319
C	4.09659	3.19577	1.57316
H	4.97536	3.85189	1.63904
H	3.24437	3.65177	2.09361
H	4.31163	2.22465	2.04164
C	3.79548	-1.22536	1.30234
N	4.11532	-0.20115	1.72579
C	3.39722	-2.51688	0.76403
H	3.80836	-2.62721	-0.24841
H	3.79383	-3.32040	1.39997
H	2.29986	-2.59233	0.73789
C	-0.88902	-3.04096	1.98367
N	0.03787	-2.77680	1.35000
C	-2.05666	-3.36087	2.78930
H	-1.86502	-4.26293	3.38639
H	-2.27968	-2.51290	3.45191
H	-2.92478	-3.52709	2.13310
C	6.20786	-0.63474	-1.11727
N	5.94790	-1.67135	-1.54957
C	6.51824	0.67606	-0.56357
H	5.92475	1.44747	-1.07355
H	6.25509	0.68796	0.50287
H	7.58714	0.89740	-0.68598
C	0.84682	1.58900	2.60319
N	0.96708	2.68234	2.25688
C	0.69666	0.21215	3.04312
H	0.77195	-0.46410	2.18241
H	1.48430	-0.03711	3.76670
H	-0.29558	0.07681	3.49642
C	-3.19776	0.22492	2.29909
N	-2.65986	-0.05812	3.27928
C	-3.87621	0.56514	1.05804
H	-3.53313	-0.11162	0.26541
H	-4.96310	0.46648	1.17645
H	-3.62624	1.59246	0.75891
C	-6.21802	1.98255	-1.36778
N	-6.38129	0.84305	-1.42943

C	-5.99162	3.41832	-1.28226
H	-6.29233	3.90259	-2.22153
H	-6.58220	3.84365	-0.45908
H	-4.92229	3.60633	-1.10028
C	-1.50552	3.53641	-0.78253
N	-2.65600	3.46513	-0.75788
C	-0.05401	3.63669	-0.80905
H	0.35381	3.39006	0.18207
H	0.36546	2.94266	-1.54893
H	0.24658	4.65869	-1.07736
C	-4.63923	-2.81818	-0.53113
N	-4.49125	-3.32949	0.49141
C	-4.81853	-2.16637	-1.82016
H	-5.27883	-2.86320	-2.53404
H	-5.46215	-1.28064	-1.71012
H	-3.83855	-1.84700	-2.19954

Nitrobenzene in Methanol

Atom	x	y	z
C	-1.29209	-3.25087	0.01586
C	0.06584	-2.94003	0.08894
C	0.52299	-1.71992	-0.39097
C	-0.40134	-0.82927	-0.92437
C	-1.75947	-1.11640	-1.00146
C	-2.20360	-2.34649	-0.52834
H	-1.64834	-4.21190	0.39376
H	0.79455	-3.62868	0.51922
H	1.57889	-1.46777	-0.33981
H	-2.45529	-0.39457	-1.42599
H	-3.27213	-2.57295	-0.57710
N	0.07871	0.45781	-1.42245
O	-0.73746	1.32020	-1.68067
O	1.27842	0.60803	-1.55925
C	1.48420	0.51270	2.28973
H	2.30995	-0.04229	1.81492
H	1.88043	1.01142	3.19468
H	0.72803	-0.21713	2.61308
O	0.86852	1.41969	1.40475
H	1.54827	1.93657	0.93661
C	-2.71378	-0.61839	2.57260
H	-3.32816	-1.52589	2.46291
H	-1.73800	-0.83517	2.09500
H	-2.53116	-0.47496	3.65699
O	-3.38659	0.45316	1.98605
H	-2.78773	1.22891	2.01894

C	3.81183	-3.52769	-0.71360
H	3.30171	-4.44827	-1.03929
H	3.66591	-2.77714	-1.51870
H	4.89641	-3.75451	-0.67253
O	3.28330	-3.13167	0.51251
H	3.75576	-2.32077	0.78981
C	4.36198	0.50546	-2.25260
H	5.24479	-0.14540	-2.33564
H	3.46535	-0.08914	-2.49382
H	4.46241	1.30303	-3.01070
O	4.32437	1.02246	-0.94441
H	3.55277	1.60947	-0.84547
C	-1.76327	3.52539	1.00895
H	-2.80351	3.87635	1.09472
H	-1.11042	4.40146	1.19465
H	-1.60265	3.19522	-0.03397
O	-1.55911	2.49927	1.93732
H	-0.66582	2.11856	1.78259
C	-4.37292	1.58495	-1.03936
H	-4.07433	1.14085	-0.07900
H	-3.54870	2.23029	-1.38906
H	-5.26813	2.21026	-0.86898
O	-4.62682	0.52240	-1.93827
H	-4.87207	0.88306	-2.79747
C	5.81659	-0.60109	1.68805
H	5.91045	-1.36704	2.47287
H	6.68322	-0.71326	1.00857
H	5.90245	0.38728	2.17872
O	4.59003	-0.77428	1.03855
H	4.50595	-0.10517	0.31851
C	1.69368	3.83623	-0.98914
H	1.01404	3.11427	-1.45820
H	1.09358	4.57528	-0.43201
H	2.25675	4.35742	-1.78191
O	2.55618	3.10449	-0.12916
H	3.16503	3.71848	0.30054
C	-5.96224	-1.52031	0.48688
H	-6.12911	-2.42128	1.09837
H	-5.29048	-0.84729	1.05193
H	-6.94314	-1.01193	0.39005
O	-5.43112	-1.91708	-0.74700
H	-5.20665	-1.10952	-1.24114

10.2 Excitation Energies for Nitrobenzene in Gas Phase and PCM Water Environment

Table S4: CASPT2//CASSCF(14,11) vertical excitation energies (eV) for the lowest singlet and triplet states of nitrobenzene at different critical points in the gas phase. Method and points taken from.[S3]

State	Structure				
	$^1(\mathrm{gs})_{\mathrm{min}}$	$^1(\mathrm{n_A}\pi^*)$	$^3(\mathrm{n_A}\pi^*)$	$^3(\pi_\mathrm{O}\pi^*)$	$(^1(\mathrm{n_A}\pi^*)/\mathrm{gs})_{\mathrm{min}}$
S_0 $^1(\mathrm{gs})_{\mathrm{min}}$	0.00	0.98	1.03	0.59	3.48
S_1 $^1(\mathrm{n_A}\pi^*)$	3.32	3.09	2.94	2.84	3.62
S_n $^1(\pi_\mathrm{O}\pi^*)$					5.63
S_2 $^1(\mathrm{n_B}\pi^*)$	3.83	4.62	4.62	4.03	6.12
S_3 $^1(\mathrm{L_B}\pi\pi^*)$	4.30	5.10	5.15	4.93	
S_4 $^1(\mathrm{L_A}\pi\pi^*)$	5.01	5.18	5.26	4.88	6.07
S_5 $^1(\pi(\pi^*)^2)$	5.53	5.90	5.73	5.65	
S_n $^1(\pi^1\pi^1\pi^*)$					6.92
T_1 $^3(\mathrm{n_A}\pi^*)$	3.12	2.93	2.76	2.66	3.49
T_2 $^3(\pi_\mathrm{O}\pi^*)$	3.28	3.15	3.20	2.99	3.66
T_3 $^3(\mathrm{L_A}\pi\pi^*)$	3.70	4.49	4.56	3.98	6.03
T_4 $^3(\mathrm{n_B}\pi^*)$	3.70	4.42	4.52	4.14	5.94
T_5 $^3(\mathrm{L_B}\pi\pi^*)$	4.11	4.99	5.04	4.59	
T_6 $^3(\pi\pi^*)$			5.31	5.91	
T_7 $^3(\mathrm{n_a^1}\pi_\mathrm{O}^1\pi^{*2})$			5.79	6.30	
T_n $^3(\pi^1\pi^1(\pi^*)^2)$					6.06

Table S5: CASPT2//CASSCF(14,11) vertical excitation energies (eV) for the lowest singlet and triplet states of nitrobenzene at different critical points including a water environment as a PCM. Method taken from[S3] and structures optimised with a PCM.

State	Structure				
	$^1(gs)_{min}$	$^1(n_A\pi^*)$	$^3(n_A\pi^*)$	$^3(\pi_O\pi^*)$	$(^1(n_A\pi^*)/gs)_{min}$
S_0 $^1(gs)_{min}$	0	0.96	0.92	0.33	3.42
S_1 $^1(n_A\pi^*)$	3.47	2.51	2.82	2.61	3.44
S_n $^1(\pi_O\pi^*)$					5.47
S_2 $^1(n_B\pi^*)$	4.06	4.23	3.91	3.68	6.03
S_3 $^1(L_B\pi\pi^*)$	4.11	4.40	4.69	4.25	
S_4 $^1(L_A\pi\pi^*)$	4.73	4.98	5.51	4.67	6.49
S_5 $^1(\pi(\pi^*)^2)$	5.71	5.16	6.61	5.29	
S_n $^1(\pi^1\pi^1\pi^*)$					7.49
T_1 $^3(n_A\pi^*)$	3.33	2.34	2.78	2.78	3.30
T_2 $^3(\pi_O\pi^*)$	3.51	2.95	2.77	2.70	4.75
T_3 $^3(L_A\pi\pi^*)$	3.57	3.94	3.80	3.58	5.52
T_4 $^3(n_B\pi^*)$	3.94	4.26	4.21	3.70	6.09
T_5 $^3(L_B\pi\pi^*)$	4.04	5.19	5.73	4.89	
T_6 $^3(\pi\pi^*)$			5.97	5.48	
T_7 $^3(n_a^1\pi_O^1\pi^{*2})$			6.46	5.92	
T_n $^3(\pi^1\pi^1(\pi^*)^2)$					6.15

References

(S1) Grubb, M. P.; Orr-Ewing, A. J.; Ashfold, M. N. R. KOALA: A program for the processing and decomposition of transient spectra. *Rev. Sci. Instrum.* 2014, *85*, 64104.

(S2) Greetham, G. M.; Donaldson, P. M.; Nation, C.; Sazanovich, I. V.; Clark, I. P.; Shaw, D. J.; Parker, A. W.; Towrie, M. A 100 kHz time-resolved multiple-probe femtosecond to second infrared absorption spectrometer. *Appl. Spectrosc.* 2016, *70*, 645–653.

(S3) Giussani, A.; Worth, G. A. Insights into the Complex Photophysics and Photochemistry of the Simplest Nitroaromatic Compound: A CASPT2//CASSCF Study on Nitrobenzene. *J. Chem. Theor. Comput.* 2017, *13*, 2777–2788.
